# Age-specific effects of a sustained cognitive activity on perceived cognitive fatigue as well as single- and dual-task treadmill walking performance

**DOI:** 10.1007/s11357-024-01452-1

**Published:** 2025-01-15

**Authors:** Martin Schlegel, Matthias Weippert, Frank Feldhege, Franziska Knaack, Thomas Mittlmeier, Sven Bruhn, Martin Behrens

**Affiliations:** 1 Institute of Sport Science, University of Rostock, Am Waldessaum 23a, 18057 Rostock, Germany; 2 Department of Traumatology, Hand and Reconstructive Surgery, University Medicine Rostock, Rostock, Germany; 3 University of Applied Sciences for Sport and Management Potsdam, Potsdam, Germany; 4 Department of Orthopaedics, University Medicine Rostock, Rostock, Germany

**Keywords:** Mental fatigue, Executive functions, Gait performance, Cognitive performance

## Abstract

During their daily lives humans are often confronted with sustained cognitive activities (SCA) leading to state fatigue, a psychobiological state characterized by a decrease in cognitive and/or motor performance and/or an increase in perception of fatigue. It was recently shown that performing SCA can impair overground dual-task gait performance in older adults, but it is currently unknown whether there is a task- and/or age-specific modulation in gait performance during treadmill walking. Therefore, the effect of a SCA on single- and dual-task treadmill walking performance was investigated in young and old adults. Using a crossover design, spatio-temporal gait parameters of 24 young and 23 older healthy participants were measured using motion capturing during single- and dual-task (including three cognitive interference tasks: word list generation, arithmetic, and Stroop-task) treadmill walking before and after SCA (30 min Stroop-task) and a control task (reading). Moreover, cognitive fatigue, wakefulness, mood, and arousal were assessed. Although the SCA induced age-specific perceptual responses, no difference was found for cognitive performance during the Stroop-task. The cognitive interference task performance (word list generation, arithmetic, and Stroop-task) during walking on the treadmill did not decrease after the SCA. Single- and dual-task gait performance (e. g., step width and step length) specifically changed after the SCA and after the reading control task in both groups. Data indicate that perceived cognitive fatigue has an impact on single- and dual-task treadmill walking performance, with task- and age-specific differences. Although no general age-specific changes in single- and dual task gait performance following SCA were identified, perceived cognitive fatigue should be considered as an intrinsic risk factor for falls.

**Supplementary Information:**

The online version contains supplementary material available at 10.1007/s11357-024-01452-1.

## Introduction

Performing a cognitive or physical task over extended periods of time is important for human life and is required during prolonged daily educational, vocational, and physical activities. The execution of sustained cognitive or motor activities inevitably leads to state fatigue, which can be defined as a psychophysiological condition characterized by a decreased cognitive or motor performance and/or an increased perception of fatigue. The acute reduction in cognitive and motor performance can be labeled as cognitive and motor performance fatigue, respectively. The increase in the cognitive and motor task-induced perception of fatigue can be categorized as perceived cognitive and motor fatigue, respectively [1–4]. It has been repeatedly demonstrated that perceived cognitive fatigue (traditionally termed subjective cognitive or mental fatigue) can increase while performing a sustained and/or intense cognitive task [5–7]. It is assumed that perceived cognitive fatigue induced by sustained cognitive tasks depends on different determinants (e.g., effort perception, arousal, affective valence), which can be quantified via scales and questionnaires. During ongoing cognitive activity, perceived cognitive fatigue and its determinants are assumed to affect the integrity of the performer and thereby contribute to changes in cognitive task performance [1, 3, 4, 7–9]. Cognitive performance fatigue, which can be objectively quantified, is characterized by a change in a cognitive performance measure over time (e.g., reaction time and/or accuracy during and/or after a cognitive task) and depends on the integrity of the central nervous system [4, 10–14]. Performing a sustained cognitive demanding activity has been shown to impair performance during subsequently executed endurance-based and motor skill-based tasks [15–21]. Moreover, we have recently found that performing a sustained response inhibition task for 90 min increased perceived cognitive fatigue as well as dual-task gait variability during overground walking in older but not younger adults [22]. Given that dual-task gait variability is associated with the risk of falls [23–25], we cautiously concluded that the susceptibility to the cognitive task-induced impairments might be an overlooked intrinsic risk factor for falls in older adults. Although it has been thereafter shown that performing different cognitive tasks for 30 min had no effect on gait measures during single-task walking on a treadmill in young and old adults [26], nothing is currently known about its effects on dual-task treadmill walking performance in these populations. In this regard, it is likely that executing a sustained cognitive task impairs dual-task but not single-task treadmill walking performance especially in older adults as this was found during overground walking [22]. Furthermore, treadmill walking was associated with a higher concentration of oxygenated hemoglobin in the prefrontal and motor cortices compared to overground walking [27] indicating an increased neural demand, which might additionally impair dual-task treadmill walking performance after a sustained cognitive task in older adults. The increased susceptibility to the cognitive task-induced impairments in old adults might be related to the age-related cognitive decline, especially of executive functions, with negative consequences for gait performance [28–30].

According to the executive function model of Diamond [31], three core executive functions exist (i.e. working memory, inhibitory control, and cognitive flexibility), which seem to be partly related to gait performance [32, 33]. Performing different executive function tasks during walking (i.e., cognitive-motor dual-task) seems also to specifically affect gait performance [34–36]. Accordingly, selectively “depleting” one of these resources with a specific sustained cognitive task might have a unique impact on different concurrent cognitive tasks and gait performance during dual-task treadmill walking.

Therefore, we investigated the effects of a sustained response inhibition task (30 min Stroop-task) on indices of perceived cognitive fatigue and cognitive performance fatigue and as well as single- and dual-task treadmill walking performance in young and old adults. In the dual-task conditions, subjects had to perform three different cognitive tasks potentially reflecting the core executive functions (i.e., word list generation task [cognitive flexibility], arithmetic task [working memory], and Stroop-task [response inhibition]). We hypothesized that the sustained response inhibition task (30 min Stroop-task) induces cognitive performance fatigue and perceived cognitive fatigue as well as exerts age-specific effects on treadmill walking performance, especially in the dual-task conditions (i.e., while performing the different concurrent cognitive tasks).

## Methods

### Participants

An a priori sample size calculation was conducted based on the effect size (ƞ_p_^2^ = 0.249) of a previously published study investigating the impact of sustained response inhibition task on gait performance during dual-task overground walking in young and old adults [22]. The sample size calculation (two-side significance alpha level of 0.05 and a power of 0.95) indicated that 16 participants per age-group (young adults aged 18 to 35 years; older adults aged 65 to 85 years) would be required. Participants were recruited via notices in the Senior Academy and via a distribution list to students at the University of Rostock. All inclusion and exclusion criteria were already communicated in the announcements. To account for potential drop-outs, 24 young (8 males; age: 25.8 ± 4.5 years; weight: 75.2 ± 11.5 kg; height: 1.80 ± 0.11 m; BMI 23.2 ± 2.1 kg/m^2^) and 23 old adults (13 males; 72.8 ± 5.1 years; 73.9 ± 9.7 kg; 1.71 ± 0.09 m; BMI 21.6 ± 2.2 kg/m^2^) without orthopedic, neurological, and cardiovascular diseases volunteered to participate in the present study. For better comparability of the two age groups, participants with a similar self-reported level of physical activity that did not exceed a maximum of 4 h per week were recruited (young: 2.7 ± 1.5 h; old: 1.9 ± 0.9 h) (see supplementary data, Tab. S1). All volunteers were informed about the experimental procedures and possible risks associated with the experiment before giving their written consent. However, participants were kept naive to the main goal of the study. The participants were instructed to refrain from intense physical and cognitive exercises as well as the consumption of alcohol and caffeine 48 h prior to the experiment. The study was conducted according to the declaration of Helsinki and was approved by the local ethics committee.

### Study design and procedures

**Fig. 1:**
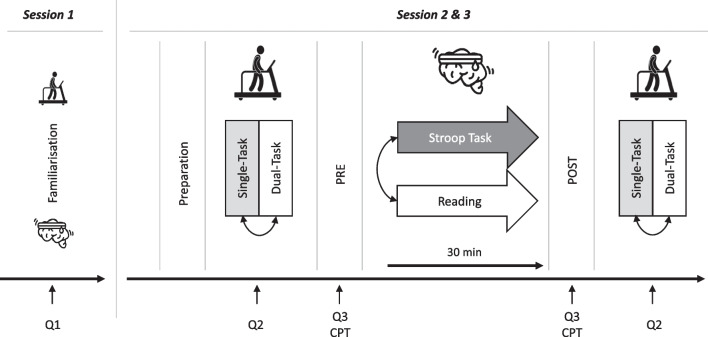
Timeline of testing procedures. Session 1: Familiarisation; determination of the individual self-selected comfortable gait speed (15 min), explanation and testing of cognitive interference tasks (Word list generation task, Arithmetic task, Stroop-task) as well as Stroop intervention task (5 min), Q1 (One-time questionnaires) = Mini-Mental State Examination (MMSE), Fall Efficacy Scale International (FES-I), Modified Fatigue Impact Scale (MFIS); Session 2 & 3: Q2 (Recurring questionnaires on dual-task interference) = visual analogue scale (VAS)—attentional focus/prioritization; Q3 (Recurring intervention questionnaires) = Profile of Mood States (POMS-F), Multidimensional Mood Questionnaire (MDMQ), German current mood scale (Aktuelle Stimmungsskala (ASTS)), Dundee Stress State Questionnaire (DSSQ), CPT (cognitive performance test) = Trail-Making Test (TMT)

Using a randomized, counterbalanced, crossover design, participants visited the laboratory on three separate occasions (Fig. 1). In the first session, they were informed about the experimental procedures and completed questionnaires (*see One-time questionnaires*). In addition, participants were familiarized with the measurement systems and the usage of the treadmill to find an individual self-selected comfortable gait speed, which was used during all walking trials. Cognitive interference tasks during walking (word list generation task, arithmetic task, and Stroop-task) as well as the Stroop-task used for the sustained cognitive intervention task were explained and carried out to avoid learning effects during the experimental sessions.

The experimental trials were separated by 7 ± 1 days and took place at the same time of the day ± 2 h. During these sessions, participants completed two randomly assigned interventions: (i) performing a Stroop-task for 30 min and (ii) reading in a free-selectable magazine for 30 min (control condition) [21, 37, 38]. Before and after each intervention, perceived fatigue, mood, arousal, wakefulness, motivation to perform the sustained cognitive activity, and cognitive performance were assessed *(see Recurring intervention questionnaires and Cognitive performance test*). Furthermore, gait performance under randomly assigned single- and dual-task conditions was also quantified before and after each intervention. In the dual-task conditions, three different concurrent attention-demanding cognitive interference tasks (word list generation task [cognitive flexibility], arithmetic task [working memory], Stroop-task [response inhibition]) were randomly performed (three times for 30 s each) during the dual-task walking trials (without explicit instructions regarding prioritization). After each completed dual-task block, perceived attentional focus/prioritization of the tasks (motor task vs. cognitive task) were retrospectively queried using a visual analogue scale (VAS) (*see Recurring questionnaires on dual-task interference*). All cognitive and motor tasks were also performed separately from each other in single-task conditions.

### Questionnaires

#### One-time questionnaires

The Mini-Mental State Examination (MMSE) was used to quantify the cognitive status and to rule out dementia or other cognitive impairments of the participants [39]. In addition, the Falls Efficacy Scale International (FES-I) was applied as a measure of fall-related self-efficacy, especially in older adults [40, 41]. The Modified Fatigue Impact Scale (MFIS) was utilized to assess the level of trait fatigue over the course of the last 7 days [42, 43].

#### Recurring intervention questionnaires

The Fatigue scale of the Profile of Mood States (POMS-F) was used to quantify perceived fatigue [44] before and after the Stroop-task and the reading control task. Beyond that, the Multidimensional Mood Questionnaire (MDMQ) was used to assess intervention-induced changes in wakefulness (awake-tired), mood (positive–negative), and arousal (calm-nervous) [45]. In addition, the German current mood scale (Aktuelle Stimmungsskala (ASTS)) including the dimensions sadness, hopelessness, tiredness, positive mood, and anger was applied [46]. Participants´ motivation to perform the sustained cognitive activity intervention was assessed using the success motivation and task interest motivation subscale of the Dundee Stress State Questionnaire (DSSQ). Subjects rated 8 items (e.g., “I wanted to succeed on the task” and “I was eager to do well.”) [47].

#### Recurring questionnaires on dual-task interference

After the completion of the randomized dual-task blocks, a VAS was used to retrospectively assess stress and the attentional focus/prioritization for the motor- and the cognitive-task during dual-task walking. Regarding the attentional focus/prioritization during dual-task walking, subjects were asked: “Indicate which task you have given more priority or attention during the simultaneous execution.” (0 = motor task; 100 = cognitive task).

### Cognitive performance test

The Trail-Making Test (TMT) [48] was applied before and after the Stroop-task and the reading control task to quantify potential intervention-related changes in cognitive performance. The TMT is used as a cumulative measure to evaluate processing speed (visuomotor abilities) and the executive functions working memory and attentional set-shifting or task-set switching, which are often summarized under the term cognitive flexibility [48–50]. In the absence of motor or sensory deficits, the TMT is sensitive to normal age-related declines in concentration, vigilance, and visuo-spatial ability that occur in later life [48], which are particularly important for maintaining gait stability.

### Gait analysis on the treadmill

Marker based gait analysis was performed with the Gait Real-time Analysis Interactive Lab (GRAIL) (Motekforce Link, Amsterdam, Netherlands). The GRAIL is an instrumented dual-belt treadmill with embedded force plates and a 180° semi-cylindrical screen on which virtual realities, images, and videos can be displayed. In addition, the GRAIL consists of a motion capture system with ten high-resolution infrared cameras (Vicon, Oxford, UK). The Lower Limb Human Body Model 2 (HBM2, Motekforce Link) with 26 retroreflective markers, was used to capture kinematic data. Marker data were recorded with Vicon Nexus (Version 2.6.0) using a sampling frequency of 250 Hz. The treadmill and the projectors were controlled with the D-Flow software (Motekforce Link, Version 3.30.1). The projectors and the semi-cylindrical screen were used for the presentation of the Stroop-task during dual-task treadmill walking. Prior the first measurement session, all participants performed a familiarisation on the GRAIL for 10–15 min. Subjects were instructed to walk in the medio-lateral middle of the dual-belt treadmill, without further instructions. All participants wore the same flat shoes at all appointments.

Initial contact and toe-off events of the feet were detected using a coordinate-based algorithm [51], which was applied using MATLAB (Mathworks, Inc., Natick, MA, USA). According to this, the anteroposterior coordinate of the foot markers relative to the displacement of the body center show a sinusoidal curve about the origin, when plotted versus time. Local maxima from a marker fixed at the toe correspond to the time where the foot lifts of the treadmill, local minima from a marker fixed at the heel correspond to the time where the foot touches the belt. We used the markers fixed at the left and right caput of the 2nd metatarsal bones and the markers fixed at the left and right center of the heels as foot markers from the Human Body Model 2 (HBM2) for analysis. As body center reference, we used HBM2 markers fixed at the left and right anterior superior iliac spine. Marker trajectories were low-pass filtered using a zero-phase digital 5th order elliptical filter with 1 dB passband ripple and 90 dB of stopband attenuation. A cut-off frequency of 10 Hz was used. Based on detected initial contact and toe-off events, gait data were split and normalized, and spatiotemporal parameters were calculated. Means and standard deviations of step width, step length, stride length, double support time, stride duration, stance duration, swing duration, stance phase, swing phase, cadence, as well as their respective coefficients of variation (CV) per trial were used for further statistical analysis. Considering the amount of measured gait parameters, we decided to reflect on the gait parameters step width, step length, and stride length as well as their CVs, whereas all other gait parameters are listed in the supplementary data.

### Cognitive interference tasks

Three different cognitive tasks related to the core executive functions (i.e., working memory, inhibitory control, and cognitive flexibility) were selected to investigate the influence of different cognitive interference tasks on dual-task gait performance before and after the sustained cognitive activity and control condition. The first cognitive interference task was a word list generation task (cognitive flexibility). During this task, participants were asked to name further terms for a given word (e.g., summer, winter, beach, etc.), which they associate with the initial term. All subjects were given the same terms in the respective condition. The number of generated words was counted and served as performance measure during this task. The second cognitive interference task was an arithmetic task (working memory). The most common numbers for subtraction are 3, 5 and 7, with increasing difficulty [52–54]. In pilot tests, we decided in favor of the number 3 [53, 54] to keep the level of difficulty between the cognitive tasks as equal as possible. It was very important to use a task that could also be executed under dual-task conditions and did not lead to cancellation. The participants were encouraged to verbalize the results of subtractions consecutively from a three-digit number between 300 and 600 as fast and correct as possible. Thus, the correctness as well as the number of subtractions could be determined. The third cognitive interference task was a visual-verbal Stroop-task (response inhibition). Fifteen congruent and incongruent color words were presented to the participants per trial (for Stroop-task explanation see *sustained cognitive activity*). Accuracy was quantified by counting the correct and incorrect verbalized answers. All trials in the single- or dual-task conditions were performed three times and lasted 30 s each to compare cognitive performance (number and/or accuracy) between them.

### Sustained cognitive activity

**Fig. 2:**
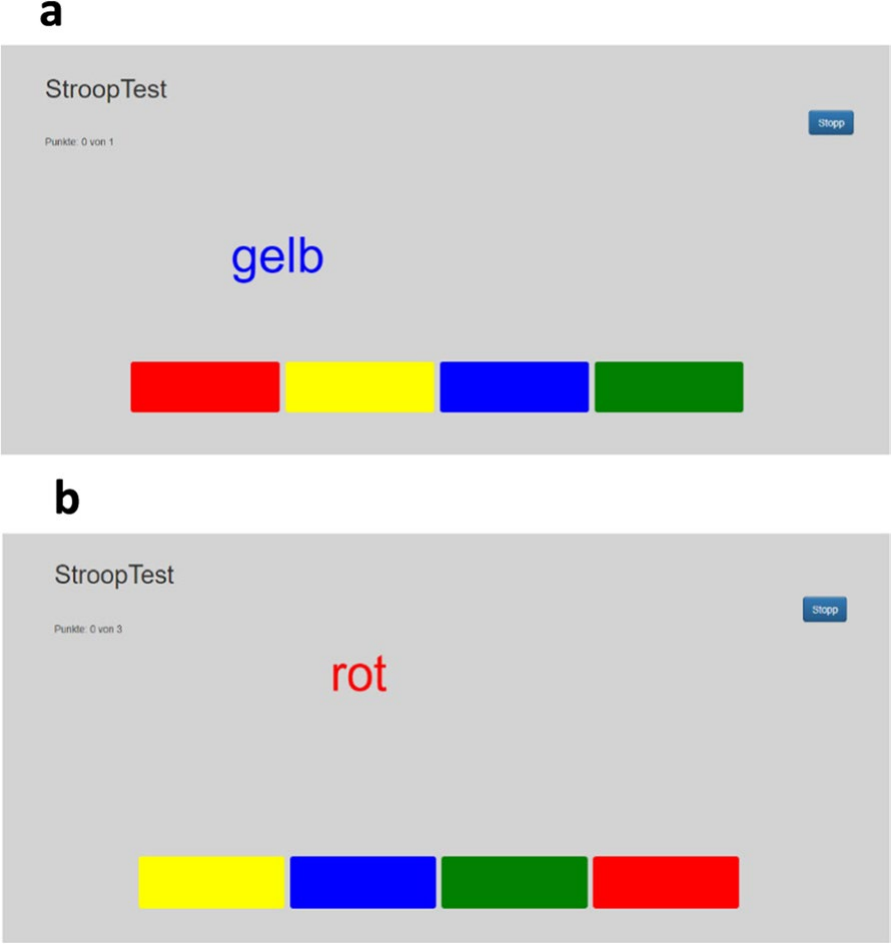
Screenshot of the Stroop intervention task (german version), which was used as sustained cognitive activity performed over 30 min during the intervention condition (**a**), incongruent task (**b**) congruent task

The participants completed two randomly assigned tasks: (i) Stroop-task (intervention task) and (ii) reading in a free-selectable magazine (control task) [21, 37, 38, 55, 56]. During the Stroop intervention task, participants were asked to perform a digital-version of the Stroop Test for 30 min, partitioned in 6 continuous blocks of 5 min on a 27-inch multi touch monitor (ACER T2 Serie; Acer Inc., Taipeh, Taiwan). This task is a commonly used laboratory response inhibition task to induce cognitive fatigue and it consists of identifying color words represented in different colors [21, 57–61]. In the congruent condition, the color word agrees with the color in which the word is displayed (Fig. 2b). In the incongruent condition, the color word and the color in which the word is displayed do not match (Fig. 2a). The ratio of congruent and incongruent trials was 25% to 75%. Maximum available processing time between the respective words was 3 s. The faster an answer was given, the faster a new task was presented. Thus, a high degree of attention could be ensured. In addition, to minimize anticipatory task adjustments, both the position of the color word on the screen and the order of the response options changed. The reaction time, number of completed tasks, as well as accuracy for each of the six blocks of 5 min (5, 10, 15, 20, 25, 30 min) were determined. Subjects were instructed to respond as quickly and accurately as possible. In the present study, we considered it important to use a cognitive task intervention duration that corresponds to situations in daily life. Therefore, in accordance with other studies revealing that performing a Stroop-task for 30 min induced perceived cognitive fatigue and, in part, had an effect on subsequent motor task performance [62–64], we have deliberately chosen the Stroop-task and its duration of 30 min. To ensure a high motivation of the participants to invest as much cognitive effort as possible in the Stroop intervention task, a financial reward (50 €) for the best performance was announced for each group [65]. Reading in a free-selectable magazine for 30 min served as the control task as done before [55, 56, 61, 66, 67].

### Statistical analyses

Data were screened for normal distribution using the Shapiro–Wilk test. Repeated measures analysis of variance (ANOVAs) with time of measurement (PRE, POST) as well as condition (reading control task, Stroop intervention task) as within-subject variables and group (young, old) as between-subjective variable was carried out for each parameter. Due to the unequal distribution of males and females between groups, sex was entered as a covariate. Effect sizes were determined using partial eta-squared (η_p_^2^) (small > 0.01, medium > 0.06, large > 0.14 effect) for the ANOVA and Cohen’s d (small > 0.2, medium > 0.5, large > 0.8 effect size) for the pairwise comparisons [68, 69]. For statistically significant main or interaction effects, Sidak-corrected post-hoc tests were conducted. The level of statistical significance was set at *P* ≤ 0.050. In addition, tendencies towards statistical significance were also interpreted (*P* ≤ 0.100). If appropriate, data are presented as mean differences (95% confidence interval). Data were analyzed using the SPSS statistical package 25.0 (SPSS Inc., USA).

## Results

### Questionnaires

#### One-time questionnaires

The scores of the MMSE (young: 29.7 ± 0.5; old: 29.0 ± 0.7; *P* = 0.001) and FES-I (young: 20.5 ± 5.0; old: 17.7 ± 1.5; *P* = 0.012) indicated that both groups were cognitively healthy and without any serious concerns about falling. Trait fatigue, assessed with the MFIS, was not different between groups (young: 17.9 ± 11.2; old: 12.8 ± 14.6; *P* = 0.194) (see supplementary data, Tab. S2).

#### Recurring intervention questionnaires

Profile of Mood States-Fatigue (POMS-F)

**Fig. 3:**
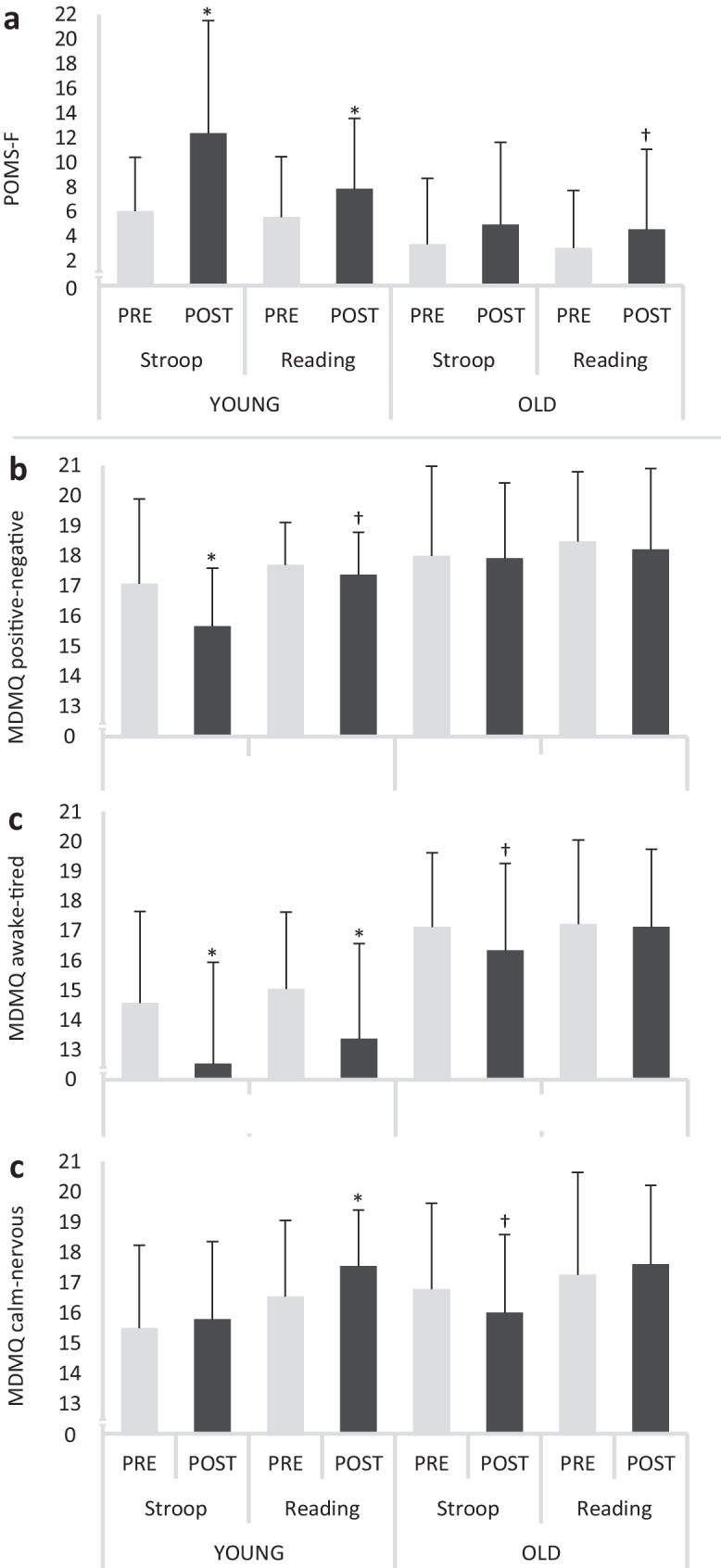
State fatigue (Profile of Mood States-Fatigue, POMS-F) (**a**), the dimensions mood (positive–negative) (**b**), wakefuleness (awake-tired) (**c**), and arousal (calm-nervous) (**d**) of the Multidimensional Mood Questionnaire (MDMQ) for the young and old participants before and after the Stroop intervention task and the reading control task. Please note that the lower the values of the respective MDMQ score, the more negative, tired, and nervous the participants felt. * *P* ≤ 0.050, † *P* ≤ 0.100

A main effect of time was found for POMS-F (*P* = 0.005, *F*_1,44_ = 8.906, ƞ_p_^2^ = 0.168). Post-hoc analysis revealed that the POMS-F score increased significantly in young adults after the Stroop intervention task (*P* = 0.001, diff.: 5.9 (2.8 to 9.0), *d* = 0.737) as well as after the reading control task (*P* = 0.016, diff.: 2.2 (0.3 to 4.1), *d* = 0.474). For the older adults, a tendency towards a significantly increased POMS-F score was observed after reading (*P* = 0.052, diff.: 1.6 (−0.4 to 3.6), *d* = 0.361) but not after performing the Stroop intervention task (*P* = 0.116, diff.: 2.0 (−1.2 to 5.2), *d* = 0.263) (Fig. 3a).

Multidimensional Mood Questionnaire (MDMQ)

A statistical tendency for a condition × time × group interaction was observed for MDMQ mood (*P* = 0.069, *F*_1,44_ = 3.474, ƞ_p_^2^ = 0.073). Post-hoc analysis revealed that only the young participants experienced a more negative mood after the Stroop intervention task (*P* = 0.011, diff.: −1.4 (−2.4 to −0.5), *d* = 0.506) and a statistical tendency towards negative mood after the reading control task (*P* = 0.088, diff.: −0.3 (−0.9 to 0.3), *d* = 0.288) (Fig. 3b).

A significant main effect of time (*P* = 0.003, *F*_1,44_ = 10.136, ƞ_p_^2^ = 0.187) and a tendency for a time × group interaction (*P* = 0.073, *F*_1,44_ = 3.382, ƞ_p_^2^ = 0.071) was found for MDMQ wakefulness. Post-hoc analysis showed that young adults felt more tired after both the Stroop intervention task (*P* = 0.003, diff.: −1.9 (−3.1 to −0.6), *d* = 0.644) as well as after reading (*P* = 0.002, diff.: −1.5 (−2.5 to −0.6), *d* = 0.648). Older adults showed a statistical tendency towards a decreased wakefulness after performing the Stroop intervention task (*P* = 0.098, diff.: −1.0 (−2.2 to 0.3), *d* = 0.284) but not after the reading control task (*P* = 0.411, diff.: −0.2 (−1.2 to 0.7), *d* = 0.050) (Fig. 3c). No main effects or interactions were detected for MDMQ arousal (calm-nervous) scores (see supplementary data, Tab. S10) (Fig. 3d).

German current mood scale (Aktuelle Stimmungsskala (ASTS))

For the dimension tiredness of the ASTS, a statistical tendency was found for the main effect time (*P* = 0.083, *F*_1,44_ = 3.151, ƞ_p_^2^ = 0.067). Furthermore, a significant time × group interaction (*P* = 0.003, *F*_1,44_ = 9.639, ƞ_p_^2^ = 0.180) and a tendency for a condition × group interaction (*P* = 0.082, *F*_1,44_ = 3.162, ƞ_p_^2^ = 0.067) were observed for ASTS tiredness. Post-hoc analysis showed significant increases in feelings of tiredness for the young adults after performing the Stroop intervention task (P = 0.001, diff.: 3.1 (1.7 to 4.4), d = 0.772) as well as after the reading control task (*P* = 0.002, diff.: 1.9 (1.0 to 2.9), *d* = 0.684). On the other hand, for the older adults, a significant increase in feelings of tiredness was found after the Stroop intervention task (*P* = 0.020, diff.: 0.9 (−0.5 to 2.3), *d* = 0.464) but not after reading (*P* = 0.373, diff.: −0.1 (−1.0 to 0.9), *d* = 0.064). A time × group interaction was found for the dimension mood of the ASTS (*P* = 0.006, *F*_1,44_ = 8.523, ƞ_p_^2^ = 0.162). Post-hoc analysis showed, only for the young adults, decreases from positive to negative mood after the Stroop intervention task (*P* = 0.001, diff.: −4.6 (−7.2 to −2.0), *d* = 0.736) as well as after reading (*P* = 0.012, diff.: −2.1 (−4.1 to −0.2), *d* = 0.504) (see supplementary data, Tab. S8).

#### Recurring questionnaires on dual-task interference

For VAS stress, a significant condition × group interaction was observed during dual-task walking while performing the Stroop-task (*P* = 0.011 *F*_1,44_ = 6.995, ƞ_p_^2^ = 0.137). Post-hoc analysis revealed no significant differences between young and older adults after both conditions for the Stroop dual-task condition. A statistical tendency for condition × time × group was found for stress during dual-task walking while performing the word list generation task (*P* = 0.089, *F*_1,44_ = 3.029, ƞ_p_^2^ = 0.064). Post-hoc analysis showed a significant reduction of stress after the Stroop intervention task for the older adults for the word-list generation task dual-task condition (*P* = 0.037, diff: −0.687 (−13.31 to −0.43), *d* = 0.357). For VAS attentional focus/prioritization, a statistical tendency for a condition × group interaction was observed for dual-task walking while performing the word list generation task (*P* = 0.088, *F*_1,44_ = 3.049, ƞ_p_^2^ = 0.065). However, post-hoc analysis revealed no differences.

## Gait parameters

In the following section, only step width, step length, and stride length recorded during single- and dual-task walking as well as their respective CVs are shown. Further gait parameters (double support time, stride duration, stance duration, swing duration, stance phase, swing phase, cadence) can be found in the supplementary data (Tab. S4 & S5). In addition, only main and/or interaction effects related to time are listed. All further statistical results of the ANOVA can also be found in the supplementary data (Tab. S10).

### Single-task gait performance

A significant main effect of time was found for step width (*P* = 0.015, *F*_1,44_ = 6.417, ƞ_p_^2^ = 0.127). For the older adults, post-hoc analysis revealed a significant decrease in step width after performing the Stroop intervention task (*P* = 0.001, diff.: −0.010 m (−0.016 m to −0.005 m), *d* = 0.807) and no reductions after reading (*P* = 0.133, diff.: −0.003 m (−0.007 m to 0.001 m), *d* = 0.267). Young adults showed statistical tendencies towards a significant decrease in step width after the Stroop intervention task (*P* = 0.093, diff.: −0.005 m (−0.010 m to 0.001 m), *d* = 0.407) as well as after reading (*P* = 0.092, diff.: −0.004 m (−0.008 m to 0.001 m), *d* = 0.496).

**Fig. 4:**
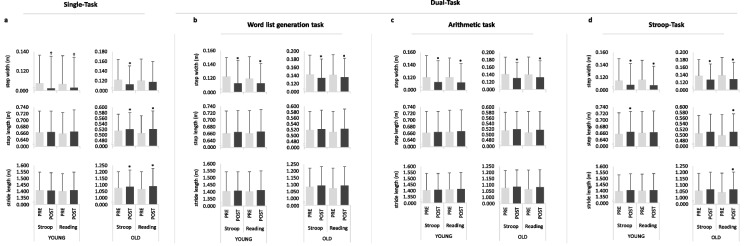
Spatio-temporal gait parameters (step width, step length, and stride length) for Single-Task (**a**) and Dual-Task: Word list generation task (**b**), Arithmetic task (**c**), and Stroop-Task (**d**) for the young and old participants recorded before (PRE) and after (POST) the interventions (Stroop intervention task (Stroop), reading control task (Reading)). * *P* ≤ 0.050, † *P* ≤ 0.100

**Fig. 5:**
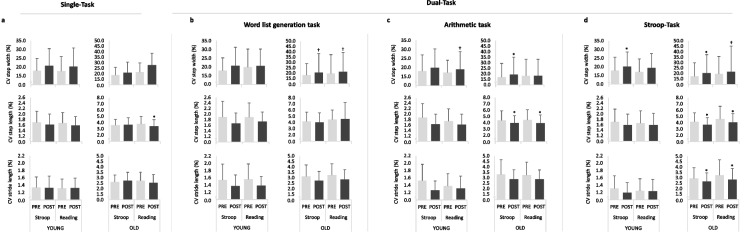
Coefficient of variation (CV) of the spatio-temporal gait parameters (step width, step length, and stride length) for Single-Task (**a**) and Dual-Task: Word list generation task (**b**), Arithmetic task (**c**), and Stroop-Task (**d**) for the young and old participants recorded before (PRE) and after (POST) the interventions (Stroop intervention task (Stroop), reading control task (Reading)). * *P* ≤ 0.050, † *P* ≤ 0.100

A significant main effect of time was found for step length (*P* = 0.012, *F*_1,44_ = 6.932, ƞ_p_^2^ = 0.136). Furthermore, a time × group interaction (*P* = 0.018, *F*_1,44_ = 6.033, ƞ_p_^2^ = 0.121) and a statistical tendency for condition × time interaction (*P* = 0.068, *F*_1,44_ = 3.494, ƞ_p_^2^ = 0.074) for step length was observed. Post-hoc analysis indicated significant increases in step length after the Stroop intervention task in the older participants (*P* = 0.013, diff.: 0.005 m (0.001 m to 0.009 m), *d* = 0.466) as well as after the reading control task (*P* = 0.000, diff.: 0.015 m (0.008 m to 0.023 m), *d* = 0.632) (Fig. 4a). For the CV_step length_ a statistical tendency for a condition × time × group interaction (*P* = 0.091, *F*_1,44_ = 2.995, ƞ_p_^2^ = 0.064) was found. For the older adults, post-hoc analysis revealed no significant reduction of CV_step length_ after performing the Stroop intervention task (*P* = 0.418, diff.: 0.092% (−0.135% to 0.320%), *d* = 0.140) but after reading (*P* = 0.007, diff.: −0.352% (−0.603% to −0.101%), *d* = 0.492) (Fig. 5a).

A significant time × group interaction was found for stride length (*P* = 0.024, *F*_1,44_ = 5.452, ƞ_p_^2^ = 0.110). Post-hoc analysis showed, similar to the step length, significant increases in stride length after the Stroop intervention task (*P* = 0.034, diff.: 0.010 m (0.001 m to 0.018 m), *d* = 0.419) and after reading (*P* = 0.000, diff.: 0.022 m (0.011 m to 0.034 m), *d* = 0.659) only for the older participants (Fig. 4a).

### Dual-task gait performance

#### Word list generation task (cognitive flexibility)

A statistical tendency was found for the main effect time for step width (*P* = 0.084, *F*_1,44_ = 3.130, ƞ_p_^2^ = 0.066). For the young adults, post-hoc analysis indicated a significant reduction after performing the Stroop intervention task (*P* = 0.000, diff.: −0.009 m (−0.014 m to −0.005 m), *d* = 0.888) as well as after the reading control task (*P* = 0.010, diff.: −0.007 m (−0.013 m to −0.002 m), *d* = 0.619). Older adults showed a similar reduction in step width after the Stroop intervention task (*P* = 0.001, diff.: −0.009 m (−0.014 m to −0.004 m), *d* = 0.782) and after the reading task (*P* = 0.035, diff.: −0.006 m (−0.011 m to 0.000 m), *d* = 0.433) (Fig. 4b). A significant main effect of time (*P* = 0.040, *F*_1,44_ = 4.490, ƞ_p_^2^ = 0.093) and a statistical tendency for a condition × time interaction (*P* = 0.076, *F*_1,44_ = 3.311, ƞ_p_^2^ = 0.070) was found for the CV_step width_. Post-hoc analysis revealed a statistical tendency towards an increased CV_step width_ after the Stroop intervention task (*P* = 0.099, diff.: 2.943% (−0.573% to 6.459%), *d* = 0.249) as well as after the reading control task (*P* = 0.055, diff.: 1.789% (−0.038% to 3.617%), *d* = 0.489) for the older adults (Fig. 5b).

#### Arithmetic task (working memory)

A significant main effect of time was found for step width (*P* = 0.001, *F*_1,44_ = 13.841, ƞ_p_^2^ = 0.239). For the young participants, post-hoc analysis showed a significant reduction after the Stroop intervention task (*P* = 0.008, diff.: −0.007 m (−0.013 m to −0.002 m), *d* = 0.737) as well as after reading (*P* = 0.001, diff.: −0.008 m (−0.012 m to −0.004 m), *d* = 0.949). These significant decreases were also observed for the older participants after the Stroop intervention task (*P* = 0.000, diff.: −0.011 m (−0.017 m to −0.006 m), *d* = 0.717) and after reading (*P* = 0.000, diff.: −0.008 m (−0.013 m to −0.004 m), *d* = 0.467) (Fig. 4c). A significant main effect of time (*P* = 0.001, *F*_1,44_ = 12.031, ƞ_p_^2^ = 0.215) as well as a statistical tendency for condition × time × group interaction (*P* = 0.090, *F*_1,44_ = 2.998, ƞ_p_^2^ = 0.064) was found for the CV_step width_. For the young adults, post-hoc analysis revealed no significant increase after the Stroop intervention task (*P* = 0.135, diff.: 1.446% (−0.467% to 3.359%), *d* = 0.412) but a statistical tendency towards an increase of the CV_step width_ after reading (*P* = 0.052, diff.: 1.630% (−0.017% to 3.277%), *d* = 0.389). For the older adults, a significant increase after the Stroop intervention task (*P* = 0.006, diff.: 2.805% (0.850% to 4.760%), *d* = 0.472) but no significant increase after reading was found (*P* = 0.680, diff.: 0.347% (−1.336% to 2.030%), *d* = 0.041).

A statistical tendency for a condition × time interaction (*P* = 0.083, *F*_1,44_ = 3.144, ƞ_p_^2^ = 0.067) was found for the CV_step length_. Post-hoc analysis revealed, only for the older adults, a significant decrease in CV_step length_ after performing the Stroop intervention task (*P* = 0.008, diff.: −0.411% (−0.710% to −0.113%), *d* = 0.451) as well as after reading (*P* = 0.001, diff.: −0.468% (−0.746% to −0.191%), *d* = 0.564) (Fig. 5c).

#### Stroop-task (response inhibition)

A significant main effect of time was found for step width (*P* = 0.004, *F*_1,44_ = 9.295, ƞ_p_^2^ = 0.174). Post-hoc analysis showed, for the young adults, a significant reduction in step width after performing the Stroop intervention task (*P* = 0.003, diff.: −0.006 m (−0.011 m to −0.002 m), *d* = 0.816) and after reading (*P* = 0.000, diff.: −0.009 m (−0.013 m to −0.004 m), *d* = 1.298). Significant reductions in step width were also observed for the older adults after the Stroop intervention task (*P* = 0.000, diff.: −0.011 m (−0.015 m to −0.007 m), *d* = 1.019) as well as after reading (*P* = 0.000, diff.: −0.011 m (−0.015 m to −0.006 m), *d* = 0.789) (Fig. 4d). A significant main effect of time (*P* = 0.023, *F*_1,44_ = 5.590, ƞ_p_^2^ = 0.113) was found for the CV_step width_. Post-hoc analysis showed for the young participants a significant increase after performing the Stroop intervention task (*P* = 0.043, diff.: 2.064% (0.071% to 4.057%), *d* = 1.151) bot not after reading (*P* = 0.165, diff.: 2.033% (−0.871% to 4.936%), *d* = 0.780). For the older participants, a significant increase after the Stroop intervention task (*P* = 0.003, diff.: 3.151% (1.114% to 5.188%), *d* = 0.485) and a statistical tendency toward an increase of the CV_step width_ after reading was found (*P* = 0.096, diff.: 2.504% (−0.464% to 5.472%), *d* = 0.236) (Fig. 5d).

A statistical tendency for a condition × time × group interaction was found for the step length (*P* = 0.051, *F*_1,44_ = 4.021, ƞ_p_^2^ = 0.084). Post-hoc analysis showed, for the young adults, a significant increase in step length after performing the Stroop intervention task (*P* = 0.048, diff.: 0.006 m (0.000 m to 0.013 m), *d* = 0.854) but not after reading (*P* = 0.361, diff.: 0.003 m (−0.003 m to 0.008 m), *d* = 0.462). For the older adults, no significant increase in step length after the Stroop intervention task (*P* = 0.155, diff.: 0.005 m (−0.002 m to 0.011 m), *d* = 0.251) but a significant increase after reading (*P* = 0.000, diff.: 0.012 m (0.006 m to 0.017 m), *d* = 0.761) was observed (Fig. 4d). A significant time × group interaction was found for the CV_step length_ (*P* = 0.020, *F*_1,44_ = 5.839, ƞ_p_^2^ = 0.117). Post-hoc analysis showed, only for the older adults, a significant reduction for the CV_step length_ after the Stroop intervention task (*P* = 0.002, diff.: −0.453% (−0.725% to −0.180%), *d* = 0.557) as well as after reading (*P* = 0.000, diff.: −0.535% (−0.806% to −0.265%), *d* = 0.649) (Fig. 5d).

A statistical tendency for condition × time × group interaction was found for the stride length (*P* = 0.085, *F*_1,44_ = 3.098, ƞ_p_^2^ = 0.066). Post-hoc analysis revealed, for the older adults, no significant increase in stride length after performing the Stroop intervention task (*P* = 0.240, diff.: 0.008 (−0.005 to 0.021), *d* = 0.225) but after reading (*P* = 0.000, diff.: 0.021 m (0.011 m to 0.030 m), *d* = 0.742) (Fig. 4d). A significant time × group interaction (*P* = 0.015, *F*_1,44_ = 6.379, ƞ_p_^2^ = 0.127) was observed for the CV_stride length_. For the older participants, post-hoc analysis indicated a significant decrease of the CV_stride length_ after the Stroop intervention task (*P* = 0.021, diff.: −0.271% (−0.499% to −0.042%), *d* = 0.391) and after reading (*P* = 0.000, diff.: −0.396% (−0.603% to −0.189%), *d* = 0.659) (Fig. 5d).

### Cognitive interference task performance

#### Single task performance

A significant time × group interaction (*P* = 0.010, *F*_1,44_ = 7.205, ƞ_p_^2^ = 0.141) and condition × time interaction (*P* = 0.042, *F*_1,44_ = 4.389, ƞ_p_^2^ = 0.091) was found for cognitive performance during the word list generation task under single-task condition. Post-hoc analysis showed, for the young adults, a significant increase in the number of words after the Stroop intervention task (*P* = 0.000, diff.: 3.097 (1.791 to 4.404), *d* = 0.683) but not after reading (*P* = 0.272, diff.: 0.535 (−0.435 to 1.505), *d* = 0.325). For the older adults, a significant increase in the number of words was found after the Stroop intervention task (*P* = 0.012, diff.: 1.746 (0.411 to 3.082), *d* = 0.304) and a significant decrease after the reading control task (*P* = 0.008, diff.: −1.362 (−2.354 to −0.371), *d* = 0.370).

#### Dual-task performance

During dual-task walking, no significant changes in cognitive interference task performance associated with the main factor time were found. The results for the other comparisons can be found in the supplementary data (Tab. S10).

### Cognitive performance measure

#### Trail Making Test (TMT)

A significant time × group interaction (*P* = 0.015, *F*_1,44_ = 6.450, ƞ_p_^2^ = 0.130) and main effect of group (*P* = 0.000, *F*_1,44_ = 42.087, ƞ_p_^2^ = 0.495) was found for the TMT Part B. Post-hoc analysis showed, for the older participants, a significant reduction of time to complete Part B of the TMT after the Stroop intervention task (*P* = 0.040, diff.: −6.001 s (−11.702 s to −0.301 s), *d* = 0.257) as well as after reading (*P* = 0.000, diff.: −11.964 s (−18.184 s to −5.744 s), *d* = 0.671). For the young participants, no significant reduction was observed after the Stroop intervention task (*P* = 0.583, diff.: −1.496 s (−6.945 s to 3.954 s), *d* = 0.185) but after reading (*P* = 0.046, diff.: −6.062 s (−12.009 s to −0.116 s), *d* = 0.920). No other main effects or interactions were found for the TMT Part B (supplementary data, Tab. S10). A statistical tendency for time × group interaction was found for the TMT (B-A) (*P* = 0.062, *F*_1,44_ = 3.662, ƞ_p_^2^ = 0.078). Post-hoc analysis showed no significant reduction after the Stroop intervention task (*P* = 0.427, diff.: −2.491 s (−8.754 s to 3.772 s), *d* = 0.055) but after reading (*P* = 0.002, diff.: −10.085 s (−16.399 s to −3.772 s), *d* = 0.563) for the older adults. For the young adults, no changes were found after both the Stroop intervention task as well as after the reading control task. No other main effects or interactions were observed for the TMT (B-A) (supplementary data, Tab. S10).

#### Stroop intervention task performance

A statistical tendency for a time × group interaction (*P* = 0.068, *F*_5,150_ = 2.102, ƞ_p_^2^ = 0.065) was found for the number of tasks. Post-hoc analysis showed, for the young participants, a significant increase in number of tasks in the period from 10 to 15 min in (*P* = 0.016, diff.: 18.135 (2.223 to 34.047), *d* = 0.642). No other significant changes were observed for the young and old participants. For the reaction time during the Stroop intervention task, a significant main effect of time (*P* = 0.005, *F*_5,150_ = 3.467, ƞ_p_^2^ = 0.104) was found. For the young adults, post-hoc analysis revealed a significant decrease in reaction time in the period from 10 to 15 min (*P* = 0.036, diff.: −0.071 s (−0.138 s to −0.003 s), *d* = 0.577), corresponding to the increased number of tasks during this time period. No further main effects nor interactions were found for reaction time as well as accuracy during the sustained Stroop intervention task (supplementary data, Tab. S10).

## Discussion

The present study has investigated the effects of a sustained response inhibition task (30 min Stroop-task) on measures of perceived cognitive fatigue and cognitive performance fatigue as well as on single- and dual-task treadmill walking performance in young and older adults. We have found that the Stroop intervention task as well as the reading control task induced changes in the subjective perceptions along with changes in gait performance during single- and dual-task treadmill walking in both age groups.

### Perceived cognitive fatigue and other perceptual responses

Age- and task-specific changes in perceived cognitive fatigue, wakefulness, mood, and arousal indicate that the sustained response inhibition task as well as the reading control task induced changes in the perceptual responses. Perceived cognitive fatigue could only be found in the young adults after the Stroop intervention task. This finding is consistent with those of other studies showing that cognitive tasks requiring response inhibition lead to perceived cognitive fatigue in young adults (21.4 ± 3.5 years) [61]. However, contrary to other studies [21, 61, 66], reading as active control-task induced also perceived cognitive fatigue in both age-groups. In this context, it has already been shown that a control task, such as reading, can induce boredom, which can also lead to changes in perceived cognitive fatigue, motivation, and the affective state [70] as well as to different oxygenation levels in the prefrontal cortex measured with functional near-infrared spectroscopy (fNIRS) [71]. Therefore, reading may not be an appropriate control task for future experiments in this field.

Similar to perceived cognitive fatigue, age-dependent changes were observed for the dimensions mood, wakefulness, and arousal measured with the MDMQ as well as the ASTS in both conditions. It is striking that young adults showed changes in both conditions, whereby the older adults only demonstrated changes after the Stroop intervention task. These results are in line with other studies that have also reported alterations in mood or arousal after sustained cognitive tasks [11, 72, 73]. In this regard, Fard & Lavender [11] also assumed that changes in mood and arousal after a sustained cognitive task might be indicators of perceived cognitive fatigue. Therefore, it seems mandatory to monitor additional dimensions such as mood, arousal, affective state, and wakefulness when comparing age-specific effects of different sustained cognitive tasks. One aspect that should not be underestimated, especially when comparing sustained cognitive tasks such as the Stroop Test and reading, is the aversive experience of boredom that emerges if a task is associated with a low cognitive load or if people have to perform unsatisfactory activities [70, 74, 75]. Thus, the novelty of the Stroop intervention task as well as the handling of the digital medium might have counteracted the emerging boredom and thus the development of perceived cognitive fatigue in the older in contrast to the younger adults. This reinforces the need to evaluate other determinants (e.g., mood, arousal, boredom, etc.) beside perceived cognitive fatigue.

### Gait performance

The results of the present study indicate that both the Stroop intervention task as well as the reading control task induced changes in treadmill gait performance during single- and dual-task walking in young and older adults.

In this regard, changes in step width and step length as well as their CVs were observed in both conditions. Older adults showed significant reductions in step width in the single-task condition after the Stroop intervention task as well as in all dual-task conditions (word list generation task, arithmetic task, Stroop-task) after both the Stroop intervention task and reading control task (Fig. 4a-d). The reduction in step width in the dual-task conditions was partly accompanied by an increase in CV_step width_. Increases in step length were only found in the single-task condition after the Stroop intervention task and the reading control task in the older adults. In the dual-task conditions, step length increased only during the concurrent Stroop-task while walking after the Stroop intervention task in the young and after the reading control task in the older adults. These results are contrary to those of a previous study [26], who has found no changes in single-task gait performance (stride length, step width, single support time, swing time, cadence, and their CVs) on a treadmill after performing three mentally demanding tasks (Psychomotor Vigilance Task, Continuous Performance Test, and Stroop Test) for a total of 30 min. The changes in gait performance recorded in this study during dual-task treadmill walking (word list generation task, arithmetic task, Stroop-task) confirm the results of Behrens et al. [22], who have found increased gait variability (speed, stride length, stance time, double support time, swing time) during dual-task (arithmetic task) overground walking after a sustained cognitive response inhibition task (Stop Signal Task) performed for 90 min.

In general, a decreased CV_step width_ was interpreted as an improved dynamic gait stability [76, 77]. Thus, the increased CV_step width_ after the Stroop intervention task as well as after the reading control task especially in the older adults during dual-task walking might indicate a deterioration of the dynamic gait stability [78, 79]. In addition, an increase in step length and stride length with a corresponding decrease in CV_step length_ and CV_stride length_ was observed in the single-task and Stroop-task dual-task condition after the Stroop intervention task in older adults. Given that gait parameters should not be interpreted in isolation [80], a reduction in step width and increase in CV_step width_ combined with an increase in stride length and decrease in CV_step length_ and CV_stride length_ might indicate a deterioration of dynamic gait stability, especially in the medio-lateral direction [81]. The resulting medio-lateral instability of gait might increase the risk of sideways falls, which can lead to hip fractures, especially in individuals aged 75 and older, and therefore pose a particular risk [82, 83].

The combined changes in stride length and step width might be due to an unfavourable shift of the attentional focus to the cognitive task, given that the cognitive interference task performance was not impaired after the Stroop intervention task and the reading control task. Therefore, it could be that the susceptibility to these negative effects might be regarded as an intrinsic modulator of gait performance and the risk of falling.

### Cognitive performance

Contrary to the assumption of a decrease in cognitive performance during and after the Stroop intervention task [15, 66, 84, 85], no reductions in cognitive performance (i.e., reaction time and accuracy) were found. Moreover, TMT performance even improved with regard to processing speed (TMT Part A) and cognitive flexibility (TMT Part B) after the Stroop intervention task as well as the reading control task in young and older adults. This is in line with the results of other studies that also used a Stroop-task performed for 30 min and more as an intervention task [58, 86–88]. In this context, Brownsberger et al. [89] and Tanaka et al. [90] reported an increased activity of the prefrontal cortex measured with EEG (β-waves) during and after a cognitive demanding task like the Stroop-task and the 2-back-task. The higher activity in the prefrontal cortex was associated with improved attention, information processing, and cognitive engagement suggesting a compensatory mechanisms to maintain cognitive performance despite the presence of perceived cognitive fatigue [91]. This might explain why no cognitive performance fatigue (i.e., an increase in reaction time or a decrease in accuracy) could be observed during the Stroop intervention task and the TMT in both age groups.

### Cognitive task performance during single- and dual-task

In contradiction to the results of other studies [22, 61, 92] and our assumption that a sustained cognitive task leads to a reduction in cognitive task performance, an improvement in the word list generation task performance (i.e., number of words) was found in both age groups under single-task conditions. The reading control task, on the other hand, led to a decrease in word list generation task performance in the older adults. This could also be related to the already discussed increased activation of the prefrontal cortex during the cognitively demanding Stroop intervention task [89, 90], which might have positively influenced cognitive task performance under single task conditions.

During the dual-task conditions, no change in cognitive interference task performance after the Stroop intervention task as well as after the reading control task was found for any of the three interference tasks (word list generation task, arithmetic task, Stroop-task) in both age groups. It can be assumed that, particularly in the dual-task conditions, task-dependent prioritisation (motor vs. cognitive task) was present, although there were no instructions regarding the focus of attention in the present study. Therefore, a shift of the attentional focus to the cognitive task (i.e., cognitive task prioritization) during walking might have occurred in order to ensure maintenance of cognitive performance during dual-task walking [93]. After the interventions, no changes in task prioritization (motor vs. cognitive task) were observed in either group regardless of condition and time. In addition, it might be assumed that treadmill walking at a self-selected constant speed is not regarded as a challenging motor task at the moment of dual-task walking and a change of prioritization (attentional focus) was therefore not considered as necessary [94]. In this context, it has already been shown that a change of attention through prioritisation occurs in challenging gait conditions or balance tasks with regard to the maintenance of postural control according to the posture-first strategy in dual-task conditions [95–98].

## Limitations

The following limitations have to be considered when interpreting the results of the present study. First, reading was used as an active control task, as done previously [21, 37, 38, 55, 56], which might have influenced the outcomes differentially compared to a more passive control task (e.g., watching a video). Second, our results seem only valid for treadmill walking with a self-selected constant walking speed and no conclusions can be drawn for overground walking and/or slower and higher gait velocities/treadmill speeds.

## Conclusion

The results of this study indicate that sustained cognitive activities, like the Stroop-task and reading for 30 min, can induce changes in perceived cognitive fatigue and related perceptual responses depending on the type of sustained cognitive task and age. Further research is needed to investigate the task-specificity with regard to the development of perceived cognitive fatigue and related perceptual qualities (e.g., boredom, sleepiness) as well as the neurophysiological changes in the involved brain areas (e.g., prefrontal cortex) in young and old adults. Data further indicate that the Stroop intervention task and the reading control task have task- and age-specific effects on gait performance, but not on cognitive performance, in younger and older adults during dual-task treadmill walking. The relevance of these findings with regard to the risk of falling requires further investigation. Furthermore, it would be of interest what effects specific training interventions (e.g., cognitive-motor dual-task training, cognitive or physical training) have on cognitive performance fatigue as well as on perceived cognitive fatigue and other perceptual qualities, and if these are capable of modulating changes in single- and dual-task gait performance after sustained cognitive tasks [99–102].

It should be noted that perceived cognitive fatigue does not always have to occur together with cognitive performance fatigue during a sustained cognitive task and that changes in subsequent motor task performance can nevertheless occur. This was also demonstrated in the present study, given that perceived cognitive fatigue resulted in changes in single- and dual-task gait performance on the treadmill especially in older adults. Therefore, the combined measurement of motor/cognitive performance fatigue and perceived motor/cognitive fatigue is recommended.

## Supplementary Information

Below is the link to the electronic supplementary material.

Supplementary file1 (DOCX 384 KB)

## Data Availability

All the data presented in the article and additional data with the corresponding statistical results can be found in the supplementary data.

## Declarations

### Proposal for a running head

Perceived cognitive fatigue and dual-task gait performance.
