# Supplementary material for: Age-specific effects of a sustained cognitive activity on perceived cognitive fatigue as well as single- and dual-task treadmill walking performance
Source: GeroScience. 2025 Jan 15;47(3):3771–89. doi: 10.1007/s11357-024-01452-1 (PMC12181590; doi:10.1007/s11357-024-01452-1)
Supplement: Supplementary file 1 — Supplementary file1 (DOCX 384 KB) [file 11357_2024_1452_MOESM1_ESM.docx]

**Supplemental Data:**

Tab.S1: Demographic characteristics of the young and old participants.

Tab.S2: Cognitive status, fall-related self-efficacy and trait fatigue.

Tab.S3: Spatio-temporal gait parameters of the young and old participants during single- and dual-task walking.

Tab.S4: Coefficient of variation (CV) for the spatio-temporal gait parameters of the young and old participants during single- and dual-task walking.

Tab.S5: Performance data of the young and old participants during the Stroop intervention task.

Tab.S6: Trail Making Test (TMT) performance data of the young and old participants before and after the interventions.

Tab.S7: Single- and dual-task performance of the young and old participants before and after the interventions.

Tab.S8: Psychological responses of the young and old participants before and after the interventions.

Tab.S9: Retrospective rating of stress and attention/prioritization during dual-task walking (visual analog scales) of young and old participants before and after the interventions.

Tab.S10: Statistical outcomes (ANOVA).

Tab.S11: Spatio-temporal gait parameters of the young and old participants during single- and dual-task walking - *manuscript-specific gait parameters*.

Tab.S12: Coefficient of variation (CV) for the spatio-temporal gait parameters of the young and old participants during single- and dual-task walking - *manuscript-specific gait parameters*.

Tab.S13: Statistical outcomes (ANOVA) - *manuscript-specific gait parameters.*

Fig.S1: Spatio-temporal gait parameters (step width, step length and stride length) and their respective coefficient of variation (CV) during single and dual-task walking.

Tab.S1. Demographic characteristics of the young and old participants.

|  |  | **YOUNG** |  | **OLD** |
| --- | --- | --- | --- | --- |
|  |  | (n = 24) |  | (n = 23) |
|  |  |  |  |  |
| Females |  | 8 (33.3%) |  | 13 (56.5%) |
| Age (yrs) |  | 25.8 ± 4.6 |  | **72.9 ± 5.2**** |
| Weight (kg) |  | 75.2 ± 11.5 |  | 73.9 ± 9.7 |
| Height (m) |  | 1.80 ± 0.11 |  | **1.71 ± 0.09**** |
| Body mass index ((kg · m^–2^) |  | 23.3 ± 2.2 |  | **21.6 ± 2.2*** |
| Physical activity (h per week) |  | 2.7 ± 1.5 |  | 1.9 ± 0.9* |
| Gait speed (m ·s^-1^) |  | 1.27 ± 0.12 |  | **0.95 ± 0.13**** |
|  |  |  |  |  |

Data are presented as mean ± standard deviation. * denotes a significant difference between groups (* *P* ≤ 0.05).

Tab.S2. Cognitive status, fall-related self-efficacy and trait fatigue.

|  |  | **YOUNG** |  | **OLD** |
| --- | --- | --- | --- | --- |
|  |  |  |  |  |
| **Cognitive Status**  Mini Mental State Examination (MMST)  **Fall-related self-efficacy**  Fall Efficacy Scale International  (FES-I)  **Trait Fatigue**  Modified Fatigue Impact Scale (MFIS) |  | 29.7 ± 0.5  20.5 ± 5.0  17.9 ± 11.2 |  | **29.0 ± 0.7****  **17.7 ± 1.5***  12.8 ± 14.6 |
|  |  |  |  |  |

Data are presented as mean ± standard deviation. * denotes a significant difference between groups (* *P* ≤ 0.05).

Tab.S3. Spatio-temporal gait parameters of the young and old participants during single- and dual-task walking.

| **Parameter** | **Condition** |  | **YOUNG** | | | | | | | | |
| --- | --- | --- | --- | --- | --- | --- | --- | --- | --- | --- | --- |
|  |  |  |  | | | | | | | | |
|  |  |  | **Pre** | | | |  | **Post** | | | |
|  |  |  |  | | | |  |  | | | |
|  |  |  | **ST** | **WLG** | **AT** | **STR** |  | **ST** | **WLG** | **ATAT** | **STR** |
|  |  |  |  |  |  |  |  |  |  |  |  |
| Step width (m) | **Stroop** |  | 0.108 ± 0.029 | 0.123 ± 0.027 | 0.121 ± 0.034 | 0.115 ± 0.035 |  | 0.102 ± 0.033 | 0.113 ± 0.033 | 0.113 ± 0.034 | 0.108 ± 0.035 |
|  | **Reading** |  | 0.107 ± 0.029 | 0.120 ± 0.032 | 0.121 ± 0.030 | 0.116 ± 0.031 |  | 0.103 ± 0.031 | 0.113 ± 0.029 | 0.112 ± 0.030 | 0.107 ± 0.031 |
|  |  |  |  |  |  |  |  |  |  |  |  |
| Step length (m) | **Stroop** |  | 0.663 ± 0.065 | 0.662 ± 0.065 | 0.662 ± 0.065 | 0.659 ± 0.064 |  | 0.664 ± 0.064 | 0.665 ± 0.063 | 0.664 ± 0.062 | 0.665 ± 0.063 |
|  | **Reading** |  | 0.660 ± 0.064 | 0.662 ± 0.066 | 0.665 ± 0.065 | 0.661 ± 0.065 |  | 0.665 ± 0.067 | 0.666 ± 0.065 | 0.667 ± 0.064 | 0.664 ± 0.065 |
|  |  |  |  |  |  |  |  |  |  |  |  |
| Stride length (m) | **Stroop** |  | 1.411 ± 0.139 | 1.406 ± 0.138 | 1.406 ± 0.138 | 1.397 ± 0.136 |  | 1.408 ± 0.137 | 1.409 ± 0.134 | 1.409 ± 0.134 | 1.406 ± 0.134 |
|  | **Reading** |  | 1.402 ± 0.135 | 1.404 ± 0.141 | 1.411 ± 0.137 | 1.402 ± 0.137 |  | 1.411 ± 0.139 | 1.413 ± 0.138 | 1.416 ± 0.136 | 1.407 ± 0.137 |
|  |  |  |  |  |  |  |  |  |  |  |  |
| Double Support time (s) | **Stroop** |  | 0.115 ± 0.014 | 0.115 ± 0.013 | 0.115 ± 0.014 | 0.114 ± 0.013 |  | 0.115 ± 0.014 | 0.116 ± 0.014 | 0.116 ± 0.015 | 0.116 ± 0.014 |
|  | **Reading** |  | 0.114 ± 0.013 | 0.115 ± 0.013 | 0.116 ± 0.013 | 0.115 ± 0.013 |  | 0.116 ± 0.014 | 0.116 ± 0.014 | 0.116 ± 0.014 | 0.115 ± 0.014 |
|  |  |  |  |  |  |  |  |  |  |  |  |
| Stride duration (s) | **Stroop** |  | 1.109 ± 0.050 | 1.106 ± 0.052 | 1.107 ± 0.053 | 1.098 ± 0.050 |  | 1.110 ± 0.052 | 1.111 ± 0.052 | 1.112 ± 0.053 | 1.107 ± 0.049 |
|  | **Reading** |  | 1.102 ± 0.051 | 1.104 ± 0.055 | 1.111 ± 0.051 | 1.102 ± 0.050 |  | 1.110 ± 0.052 | 1.111 ± 0.051 | 1.115 ± 0.050 | 1.105 ± 0.048 |
|  |  |  |  |  |  |  |  |  |  |  |  |
| Stand duration (s) | **Stroop** |  | 0.670 ± 0.034 | 0.668 ± 0.035 | 0.669 ± 0.036 | 0.664 ± 0.034 |  | 0.671 ± 0.036 | 0.671 ± 0.037 | 0.672 ± 0.038 | 0.669 ± 0.034 |
|  | **Reading** |  | 0.666 ± 0.034 | 0.667 ± 0.036 | 0.671 ± 0.035 | 0.666 ± 0.034 |  | 0.670 ± 0.036 | 0.671 ± 0.035 | 0.673 ± 0.035 | 0.668 ± 0.033 |
|  |  |  |  |  |  |  |  |  |  |  |  |
| Swing duration (s) | **Stroop** |  | 0.440 ± 0.021 | 0.438 ± 0.021 | 0.438 ± 0.021 | 0.435 ± 0.021 |  | 0.440 ± 0.021 | 0.440 ± 0.020 | 0.440 ± 0.020 | 0.438 ± 0.020 |
|  | **Reading** |  | 0.437± 0.022 | 0.438 ± 0.023 | 0.440 ± 0.021 | 0.436 ± 0.021 |  | 0.439 ± 0.022 | 0.440 ± 0.021 | 0.442 ± 0.020 | 0.437 ± 0.020 |
|  |  |  |  |  |  |  |  |  |  |  |  |
| Stand phase (%) | **Stroop** |  | 60.37 ± 1.05 | 60.36 ± 0.93 | 60.42 ± 0.98 | 60.41 ± 0.97 |  | 60.39 ± 1.07 | 60.39 ± 1.01 | 60.41 ± 1.04 | 60.42 ± 1.03 |
|  | **Reading** |  | 60.37 ± 1.01 | 60.37 ± 0.95 | 60.39 ± 0.97 | 60.42 ± 0.97 |  | 60.42 ± 1.02 | 60.39 ± 1.03 | 60.37 ± 1.05 | 60.41 ± 1.04 |
|  |  |  |  |  |  |  |  |  |  |  |  |
| Swing phase (%) | **Stroop** |  | 39.63 ± 1.04 | 39.64 ± 0.93 | 39.59 ± 0.98 | 39.59 ± 0.97 |  | 39.61 ± 1.06 | 39.61 ± 1.01 | 39.59 ± 1.04 | 39.58 ± 1.03 |
|  | **Reading** |  | 39.63 ± 1.00 | 39.63 ± 0.95 | 39.61 ± 0.97 | 39.58 ± 0.97 |  | 39.59 ± 1.02 | 39.61 ± 1.03 | 39.63 ± 1.05 | 39.59 ± 1.03 |
|  |  |  |  |  |  |  |  |  |  |  |  |
| Cadence (steps/ min) | **Stroop** |  | 54.22 ± 2.54 | 54.39 ± 2.62 | 54.31 ± 2.65 | 54.75 ± 2.61 |  | 54.18 ± 2.61 | 54.12 ± 2.60 | 54.11 ± 2.68 | 54.32 ± 2.48 |
|  | **Reading** |  | 54.55 ± 2.63 | 54.48 ± 2.81 | 54.15 ± 2.60 | 54.58 ± 2.59 |  | 54.22 ± 2.66 | 54.12 ± 2.54 | 53.94 ± 2.46 | 54.40 ± 2.42 |
|  |  |  |  |  |  |  |  |  |  |  |  |
| **Parameter** | **Condition** |  | ***OLD*** | | | | | | | | |
|  |  |  |  | | | | | | | | |
|  |  |  | **Pre** | | | |  | **Post** | | | |
|  |  |  |  | | | |  |  | | | |
|  |  |  | **ST** | **WLG** | **AT** | **STR** |  | **ST** | **WLG** | **AT** | **STR** |
|  |  |  |  |  |  |  |  |  |  |  |  |
| Step width (m) | **Stroop** |  | 0.122 ± 0.042 | 0.143 ± 0.047 | 0.141 ± 0.046 | 0.138 ± 0.043 |  | 0.113 ± 0.038 | 0.134 ± 0.047 | 0.131 ± 0.044 | 0.127 ± 0.042 |
|  | **Reading** |  | 0.121 ± 0.044 | 0.142 ± 0.050 | 0.141 ± 0.047 | 0.139 ± 0.047 |  | 0.118 ± 0.042 | 0.136 ± 0.047 | 0.133 ± 0.045 | 0.129 ± 0.045 |
|  |  |  |  |  |  |  |  |  |  |  |  |
| Step length (m) | **Stroop** |  | 0.516 ± 0.060 | 0.520 ± 0.063 | 0.516 ± 0.066 | 0.507 ± 0.064 |  | 0.521 ± 0.060 | 0.523 ± 0.065 | 0.520 ± 0.065 | 0.512 ± 0.063 |
|  | **Reading** |  | 0.508 ± 0.062 | 0.513 ± 0.070 | 0.508 ± 0.077 | 0.499 ± 0.073 |  | 0.522 ± 0.066 | 0.524 ± 0.067 | 0.518 ± 0.067 | 0.512 ± 0.065 |
|  |  |  |  |  |  |  |  |  |  |  |  |
| Stride length (m) | **Stroop** |  | 1.077 ± 0.127 | 1.086 ± 0.136 | 1.077 ± 0.139 | 1.058 ± 0.135 |  | 1.087 ± 0.130 | 1.095 ± 0.140 | 1.086 ± 0.136 | 1.067 ± 0.135 |
|  | **Reading** |  | 1.070 ± 0.135 | 1.076 ± 0.147 | 1.067 ± 0.154 | 1.046 ± 0.149 |  | 1.092 ± 0.137 | 1.095 ± 0.140 | 1.083 ± 0.140 | 1.068 ± 0.137 |
|  |  |  |  |  |  |  |  |  |  |  |  |
| Double Support time (s) | **Stroop** |  | 0.138 ± 0.022 | 0.137 ± 0.023 | 0.136 ± 0.022 | 0.134 ± 0.023 |  | 0.139 ± 0.020 | 0.137 ± 0.026 | 0.138 ± 0.022 | 0.135 ± 0.021 |
|  | **Reading** |  | 0.135 ± 0.021 | 0.135 ± 0.022 | 0.133 ± 0.022 | 0.131 ± 0.022 |  | 0.137 ± 0.020 | 0.138 ± 0.022 | 0.136 ± 0.022 | 0.135 ± 0.020 |
|  |  |  |  |  |  |  |  |  |  |  |  |
| Stride duration (s) | **Stroop** |  | 1.139 ± 0.087 | 1.148 ± 0.100 | 1.139 ± 0.094 | 1.122 ± 0.101 |  | 1.150 ± 0.085 | 1.157 ± 0.101 | 1.150 ± 0.097 | 1.130 ± 0.091 |
|  | **Reading** |  | 1.132 ± 0.090 | 1.137 ± 0.100 | 1.126 ± 0.098 | 1.107 ± 0.099 |  | 1.155 ± 0.089 | 1.157 ± 0.097 | 1.145 ± 0.092 | 1.130 ± 0.088 |
|  |  |  |  |  |  |  |  |  |  |  |  |
| Stand duration (s) | **Stroop** |  | 0.707 ± 0.063 | 0.711 ± 0.071 | 0.705 ± 0.067 | 0.695 ± 0.072 |  | 0.714 ± 0.060 | 0.715 ± 0.073 | 0.714 ± 0.069 | 0.700 ± 0.066 |
|  | **Reading** |  | 0.701 ± 0.063 | 0.704 ± 0.070 | 0.696 ± 0.070 | 0.684 ± 0.069 |  | 0.715 ± 0.062 | 0.716 ± 0.068 | 0.708 ± 0.066 | 0.701 ± 0.062 |
|  |  |  |  |  |  |  |  |  |  |  |  |
| Swing duration (s) | **Stroop** |  | 0.432 ± 0.028 | 0.436 ± 0.032 | 0.434 ± 0.029 | 0.426 ± 0.032 |  | 0.436 ± 0.028 | 0.441 ± 0.031 | 0.437 ± 0.030 | 0.430 ± 0.028 |
|  | **Reading** |  | 0.431 ± 0.031 | 0.433 ± 0.032 | 0.430 ± 0.031 | 0.423 ± 0.032 |  | 0.440 ± 0.031 | 0.440 ± 0.032 | 0.436 ± 0.029 | 0.430 ± 0.030 |
|  |  |  |  |  |  |  |  |  |  |  |  |
| Stand phase (%) | **Stroop** |  | 62.02 ± 1.18 | 61.91 ± 1.14 | 61.87 ± 1.12 | 61.92 ± 1.20 |  | 62.04 ± 1.08 | 61.76 ± 1.46 | 61.95 ± 1.12 | 61.90 ± 1.10 |
|  | **Reading** |  | 61.91 ± 1.23 | 61.86 ± 1.18 | 61.72 ± 1.18 | 61.76 ± 1.17 |  | 61.87 ± 1.13 | 61.89 ± 1.13 | 61.84 ± 1.18 | 61.93 ± 1.13 |
|  |  |  |  |  |  |  |  |  |  |  |  |
| Swing phase (%) | **Stroop** |  | 37.98 ± 1.19 | 38.09 ± 1.14 | 38.14 ± 1.14 | 38.08 ± 1.19 |  | 37.97 ± 1.08 | 38.25 ± 1.47 | 38.07 ± 1.12 | 38.10 ± 1.10 |
|  | **Reading** |  | 38.10 ± 1.23 | 38.15 ± 1.18 | 38.27 ± 1.19 | 38.25 ± 1.16 |  | 38.14 ± 1.13 | 38.11 ± 1.13 | 38.17 ± 1.17 | 38.07 ± 1.13 |
|  |  |  |  |  |  |  |  |  |  |  |  |
| Cadence (steps/ min) | **Stroop** |  | 52.99 ± 3.95 | 52.69 ± 4.58 | 53.04 ± 4.26 | 53.94 ± 4.85 |  | 52.46 ± 3.79 | 52.30 ± 4.42 | 52.54 ± 4.34 | 53.44 ± 4.19 |
|  | **Reading** |  | 53.38 ± 4.22 | 53.18 ± 4.53 | 53.70 ± 4.61 | 54.65 ± 4.82 |  | 52.29 ± 3.95 | 52.24 ± 4.18 | 52.75 ± 4.04 | 53.41 ± 4.06 |
|  |  |  |  |  |  |  |  |  |  |  |  |

Data are presented as mean ± standard deviation. ST, Single-Task; WLG, Word list generation task; AT, Arithmetic task; STR, Stroop-task.

Tab.S4. Coefficient of variation (CV) for the spatio-temporal gait parameters of the young and old participants during single- and dual-task walking.

| **Parameter** | **Condition** |  | **YOUNG** | | | | | | | | |
| --- | --- | --- | --- | --- | --- | --- | --- | --- | --- | --- | --- |
|  |  |  |  | | | | | | | | |
|  |  |  | **Pre** | | | |  | **Post** | | | |
|  |  |  |  | | | |  |  | | | |
|  |  |  | **ST** | **WLG** | **AT** | **STR** |  | **ST** | **WLG** | **AT** | **STR** |
|  |  |  |  |  |  |  |  |  |  |  |  |
| CV _Step width_ (%) | **Stroop** |  | 17.94 ± 7.06 | 17.98 ± 7.28 | 18.03 ± 8.95 | 17.88 ± 7.80 |  | 20.87 ± 9.68 | 20.80 ± 10.54 | 19.88 ± 10.41 | 20.19 ± 8.40 |
|  | **Reading** |  | 17.72 ± 8.32 | 19.94 ± 10.36 | 17.26 ± 6.79 | 17.10 ± 7.52 |  | 20.29 ± 10.68 | 20.67 ± 9.63 | 19.10 ± 9.68 | 19.43 ± 8.23 |
|  |  |  |  |  |  |  |  |  |  |  |  |
| CV _Step length_ (%) | **Stroop** |  | 1.70 ± 0.40 | 1.90 ± 0.56 | 1.87 ± 0.51 | 1.72 ± 0.47 |  | 1.62 ± 0.39 | 1.67 ± 0.38 | 1.63 ± 0.37 | 1.60 ± 0.40 |
|  | **Reading** |  | 1.68 ± 0.38 | 1.89 ± 0.51 | 1.74 ± 0.46 | 1.66 ± 0.46 |  | 1.59 ± 0.32 | 1.73 ± 0.35 | 1.61 ± 0.38 | 1.60 ± 0.42 |
|  |  |  |  |  |  |  |  |  |  |  |  |
| CV _Stride length_ (%) | **Stroop** |  | 1.34 ± 0.29 | 1.54 ± 0.45 | 1.53 ± 0.44 | 1.31 ± 0.35 |  | 1.33 ± 0.32 | 1.37 ± 0.31 | 1.27 ± 0.25 | 1.20 ± 0.27 |
|  | **Reading** |  | 1.32 ± 0.25 | 1.57 ± 0.42 | 1.38 ± 0.34 | 1.24 ± 0.33 |  | 1.32 ± 0.28 | 1.38 ± 0.25 | 1.31 ± 0.34 | 1.24 ± 0.32 |
|  |  |  |  |  |  |  |  |  |  |  |  |
| CV _Double Support time_ (%) | **Stroop** |  | 4.87 ± 0.86 | 5.15 ± 1.06 | 4.73 ± 0.84 | 4.68 ± 0.80 |  | 5.13 ± 1.00 | 4.89 ± 0.88 | 4.84 ± 1.17 | 4.56 ± 0.95 |
|  | **Reading** |  | 5.11 ± 1.12 | 4.97 ± 1.03 | 4.77 ± 0.81 | 4.59 ± 0.98 |  | 4.83 ± 1.01 | 5.03 ± 0.89 | 4.81 ± 0.73 | 4.54 ± 0.82 |
|  |  |  |  |  |  |  |  |  |  |  |  |
| CV _Stride duration_ (%) | **Stroop** |  | 0.99 ± 0.27 | 1.14 ± 0.31 | 1.17 ± 0.27 | 1.01 ± 0.20 |  | 1.04 ± 0.25 | 1.13 ± 0.20 | 1.05 ± 0.18 | 0.97 ± 0.22 |
|  | **Reading** |  | 1.06 ± 0.17 | 1.15 ± 0.27 | 1.05 ± 0.20 | 1.03 ± 0.27 |  | 1.00 ± 0.18 | 1.18 ± 0.26 | 1.11 ± 0.26 | 0.97 ± 0.19 |
|  |  |  |  |  |  |  |  |  |  |  |  |
| CV _Stand duration_ (%) | **Stroop** |  | 1.47 ± 0.29 | 1.58 ± 0.39 | 1.56 ± 0.35 | 1.45 ± 0.28 |  | 1.52 ± 0.38 | 1.61 ± 0.28 | 1.49 ± 0.27 | 1.39 ± 0.29 |
|  | **Reading** |  | 1.52 ± 0.25 | 1.62 ± 0.35 | 1.52 ± 0.30 | 1.47 ± 0.37 |  | 1.44 ± 0.24 | 1.63 ± 0.34 | 1.48 ± 0.30 | 1.41 ± 0.24 |
|  |  |  |  |  |  |  |  |  |  |  |  |
| CV _Swing duration_ (%) | **Stroop** |  | 1.32 ± 0.32 | 1.46 ± 0.40 | 1.47 ± 0.40 | 1.33 ± 0.30 |  | 1.19 ± 0.35 | 1.42 ± 0.30 | 1.31 ± 0.29 | 1.27 ± 0.33 |
|  | **Reading** |  | 1.26 ± 0.34 | 1.42 ± 0.36 | 1.35 ± 0.30 | 1.34 ± 0.39 |  | 1.22 ± 0.25 | 1.40 ± 0.31 | 1.36 ± 0.30 | 1.31 ± 0.29 |
|  |  |  |  |  |  |  |  |  |  |  |  |
| CV _Stand phase_ (%) | **Stroop** |  | 0.80 ± 0.15 | 0.88 ± 0.18 | 0.86 ± 0.18 | 0.81 ± 0.17 |  | 0.82 ± 0.18 | 0.85 ± 0.14 | 0.81 ± 0.16 | 0.78 ± 0.18 |
|  | **Reading** |  | 0.83 ± 0.16 | 0.86 ± 0.18 | 0.85 ± 0.16 | 0.82 ± 0.19 |  | 0.77 ± 0.12 | 0.85 ± 0.16 | 0.81 ± 0.15 | 0.78 ± 0.13 |
|  |  |  |  |  |  |  |  |  |  |  |  |
| CV _Swing phase_ (%) | **Stroop** |  | 1.22 ± 0.23 | 1.35 ± 0.32 | 1.33 ± 0.29 | 1.23 ± 0.25 |  | 1.23 ± 0.27 | 1.31 ± 0.23 | 1.24 ± 0.25 | 1.20 ± 0.25 |
|  | **Reading** |  | 1.27 ± 0.26 | 1.32 ± 0.31 | 1.29 ± 0.26 | 1.26 ± 0.32 |  | 1.17 ± 0.21 | 1.30 ± 0.26 | 1.24 ± 0.26 | 1.18 ± 0.21 |
|  |  |  |  |  |  |  |  |  |  |  |  |
| CV _Cadence_ (%) | **Stroop** |  | 1.00 ± 0.26 | 1.15 ± 0.32 | 1.18 ± 0.28 | 1.03 ± 0.22 |  | 1.03 ± 0.24 | 1.17 ± 0.23 | 1.07 ± 0.21 | 1.04 ± 0.46 |
|  | **Reading** |  | 1.05 ± 0.17 | 1.17 ± 0.29 | 1.05 ± 0.22 | 1.02 ± 0.26 |  | 0.98 ± 0.20 | 1.16 ± 0.26 | 1.10 ± 0.27 | 0.96 ± 0.19 |
|  |  |  |  |  |  |  |  |  |  |  |  |
|  |  |  |  |  |  |  |  |  |  |  |  |
| **Parameter** | **Condition** |  | ***OLD*** | | | | | | | | |
|  |  |  |  | | | | | | | | |
|  |  |  | **Pre** | | | |  | **Post** | | | |
|  |  |  |  | | | |  |  | | | |
|  |  |  | **ST** | **WLG** | **AT** | **STR** |  | **ST** | **WLG** | **AT** | **STR** |
|  |  |  |  |  |  |  |  |  |  |  |  |
| CV _Step width_ (%) | **Stroop** |  | 18.81 ± 12.97 | 18.10 ± 10.83 | 16.94 ± 12.48 | 17.43 ± 12.49 |  | 20.93 ± 15.22 | 20.57 ± 17.83 | 19.33 ± 15.86 | 20.33 ± 16.97 |
|  | **Reading** |  | 21.44 ± 15.92 | 19.46 ± 17.87 | 18.18 ± 14.87 | 19.45 ± 16.18 |  | 27.90 ± 47.99 | 21.31 ± 18.13 | 18.30 ± 14.98 | 21.65 ± 23.60 |
|  |  |  |  |  |  |  |  |  |  |  |  |
| CV _Step length_ (%) | **Stroop** |  | 3.58 ± 0.95 | 4.19 ± 1.48 | 4.37 ± 1.53 | 4.16 ± 1.42 |  | 3.67 ± 1.13 | 4.01 ± 1.57 | 3.96 ± 1.16 | 3.68 ± 1.14 |
|  | **Reading** |  | 3.80 ± 1.23 | 4.50 ± 1.53 | 4.44 ± 1.63 | 4.61 ± 2.02 |  | 3.43 ± 1.04 | 4.57 ± 2.67 | 3.94 ± 1.31 | 4.05 ± 1.42 |
|  |  |  |  |  |  |  |  |  |  |  |  |
| CV _Stride length_ (%) | **Stroop** |  | 2.63 ± 0.62 | 3.13 ± 1.05 | 3.31 ± 1.36 | 2.95 ± 1.01 |  | 2.76 ± 0.76 | 2.74 ± 0.86 | 2.89 ± 0.86 | 2.67 ± 0.79 |
|  | **Reading** |  | 2.75 ± 0.78 | 3.27 ± 1.01 | 3.26 ± 1.18 | 3.24 ± 1.43 |  | 2.55 ± 0.75 | 2.86 ± 0.89 | 2.88 ± 0.85 | 2.83 ± 1.04 |
|  |  |  |  |  |  |  |  |  |  |  |  |
| CV _Double Support time_ (%) | **Stroop** |  | 7.08 ± 1.68 | 7.03 ± 1.54 | 7.61 ± 1.65 | 7.22 ± 1.56 |  | 7.22 ± 2.02 | 7.12 ± 2.06 | 7.60 ± 1.73 | 6.49 ± 1.57 |
|  | **Reading** |  | 7.63 ± 2.19 | 7.65 ± 1.73 | 7.78 ± 1.68 | 7.32 ± 1.69 |  | 7.11 ± 1.82 | 7.64 ± 1.88 | 7.60 ± 2.06 | 6.91 ± 1.32 |
|  |  |  |  |  |  |  |  |  |  |  |  |
| CV _Stride duration_ (%) | **Stroop** |  | 1.84 ± 0.42 | 2.04 ± 0.57 | 2.00 ± 0.53 | 1.78 ± 0.36 |  | 1.91 ± 0.54 | 1.91 ± 0.43 | 1.91 ± 0.56 | 1.61 ± 0.35 |
|  | **Reading** |  | 1.85 ± 0.52 | 2.01 ± 0.53 | 1.97 ± 0.48 | 1.86 ± 0.67 |  | 1.72 ± 0.50 | 1.87 ± 0.53 | 1.91 ± 0.50 | 1.70 ± 0.32 |
|  |  |  |  |  |  |  |  |  |  |  |  |
| CV _Stand duration_ (%) | **Stroop** |  | 2.53 ± 0.55 | 2.73 ± 0.76 | 2.76 ± 0.69 | 2.48 ± 0.53 |  | 2.54 ± 0.61 | 2.79 ± 1.03 | 2.62 ± 0.64 | 2.18 ± 0.49 |
|  | **Reading** |  | 2.61 ± 0.75 | 2.79 ± 0.71 | 2.76 ± 0.54 | 2.58 ± 0.84 |  | 2.39 ± 0.64 | 2.64 ± 0.70 | 2.69 ± 0.70 | 2.33 ± 0.49 |
|  |  |  |  |  |  |  |  |  |  |  |  |
| CV _Swing duration_ (%) | **Stroop** |  | 2.44 ± 0.57 | 2.98 ± 0.79 | 3.01 ± 0.73 | 2.61 ± 0.66 |  | 2.49 ± 0.73 | 3.14 ± 1.26 | 2.75 ± 0.67 | 2.41 ± 0.67 |
|  | **Reading** |  | 2.54 ± 0.74 | 2.88 ± 0.69 | 2.87 ± 0.66 | 2.65 ± 0.86 |  | 2.31 ± 0.70 | 2.83 ± 0.79 | 2.74 ± 0.74 | 2.43 ± 0.63 |
|  |  |  |  |  |  |  |  |  |  |  |  |
| CV _Stand phase_ (%) | **Stroop** |  | 1.37 ± 0.34 | 1.58 ± 0.40 | 1.61 ± 0.38 | 1.44 ± 0.31 |  | 1.37 ± 0.44 | 1.76 ± 0.87 | 1.57 ± 0.36 | 1.32 ± 0.33 |
|  | **Reading** |  | 1.38 ± 0.39 | 1.64 ± 0.40 | 1.62 ± 0.37 | 1.45 ± 0.38 |  | 1.33 ± 0.36 | 1.57 ± 0.38 | 1.53 ± 0.40 | 1.34 ± 0.32 |
|  |  |  |  |  |  |  |  |  |  |  |  |
| CV _Swing phase_ (%) | **Stroop** |  | 2.21 ± 0.60 | 2.54 ± 0.65 | 2.62 ± 0.63 | 2.32 ± 0.56 |  | 2.23 ± 0.77 | 2.84 ± 1.12 | 2.55 ± 0.65 | 2.14 ± 0.58 |
|  | **Reading** |  | 2.29 ± 0.67 | 2.65 ± 0.72 | 2.62 ± 0.62 | 2.36 ± 0.65 |  | 2.19 ± 0.63 | 2.56 ± 0.61 | 2.51 ± 0.73 | 2.19 ± 0.57 |
|  |  |  |  |  |  |  |  |  |  |  |  |
| CV _Cadence_ (%) | **Stroop** |  | 1.83 ± 0.41 | 2.05 ± 0.57 | 2.00 ± 0.51 | 1.76 ± 0.36 |  | 1.85 ± 0.51 | 1.95 ± 0.47 | 1.95 ± 0.72 | 1.60 ± 0.35 |
|  | **Reading** |  | 1.85 ± 0.56 | 2.05 ± 0.54 | 1.96 ± 0.46 | 1.87 ± 0.75 |  | 1.70 ± 0.49 | 1.89 ± 0.55 | 1.93 ± 0.53 | 1.70 ± 0.35 |
|  |  |  |  |  |  |  |  |  |  |  |  |

CV, coefficient of variation. ST, Single-Task; WLG, Word list generation task; AT, Arithmetic task; STR, Stroop-task. Data are presented as mean ± standard deviation.

Tab.S5. Performance data of the young and old participants during the Stroop intervention task.

| **Parameter** |  | **5 min** | | **10 min** | **15 min** | **20 min** | **25 min** | **30 min** | **Total** |
| --- | --- | --- | --- | --- | --- | --- | --- | --- | --- |
|  |  |  | |  |  |  |  |  |  |
|  |  | ***YOUNG*** | | | | | | | |
|  |  |  | | | | | | | |
| Number of tasks |  | 280.19 ± 43.36 | | 278.81 ± 45.25 | 295.86 ± 40.52 | 296.38 ± 38.49 | 302.00 ± 36.11 | 306.80 ± 41.32 | 293.34 ± 10.45 |
|  |  |  | |  |  |  |  |  |  |
| Reaction time (s) |  | 1.05 ± 0.16 | | 1.05 ± 0.17 | 0.99 ± 0.15 | 0.98 ± 0.15 | 0.96 ± 0.13 | 0.95 ± 0.14 | 0.99 ± 0.14 |
|  |  |  | |  |  |  |  |  |  |
| Accuracy (%) |  | 98.49 ± 2.27 | | 98.59 ± 2.09 | 98.74 ± 1.88 | 98.66 ± 1.50 | 99.08 ± 0.85 | 98.77 ± 1.59 | 98.77 ± 1.06 |
|  |  |  | |  |  |  |  |  |  |
|  |  | ***OLD*** | | | | | | | |
|  |  |  | | | | | | | |
| Number of tasks |  | 211.62 ± 32.29 | | 218.15 ± 25.34 | 221.23 ± 30.13 | 226.92 ± 29.61 | 227.46 ± 31.33 | 229.77 ± 33.22 | 222.53 ± 6.27 |
|  |  |  | |  |  |  |  |  |  |
| Reaction time (s) |  | 1.21 ± 0.17 | | 1.16 ± 0.14 | 1.15 ± 0.17 | 1.12 ± 0.15 | 1.12 ± 0.16 | 1.11 ± 0.17 | 1.14 ± 0.15 |
|  |  |  | |  |  |  |  |  |  |
| Accuracy (%) |  | 98.74 ± 1.90 | | 99.18 ± 1.35 | 99.23 ± 0.97 | 99.45 ± 0.70 | 98.91 ± 1.39 | 98.99 ± 1.37 | 99.10 ± 0.92 |
|  |  |  | |  |  |  |  |  |  |
|  |  | |  |  |  |  |  |  |  |

Data are presented in 6 blocks and the total number of tasks over 30 min as mean ± standard deviation.

Tab.S6. Trail Making Test (TMT) performance data of the young and old participants before and after the interventions.

| **Parameter** | **Condition** |  | **YOUNG** | |  | **OLD** | |  |
| --- | --- | --- | --- | --- | --- | --- | --- | --- |
|  |  |  |  | |  |  | |  |
|  |  |  | **Pre** | **Post** |  | **Pre** | **Post** |  |
|  |  |  |  |  |  |  |  |  |
| **TMT-A (s)** | **Stroop** |  | 15.99 ± 4.07 | 14.80 ± 3.54 |  | 30.43 ± 10.19 | 27.16 ± 5.60 |  |
|  | **Reading** |  | 16.49 ± 5.00 | 14.06 ± 3.51 |  | 29.06 ± 10.62 | 27.12 ± 9.83 |  |
|  |  |  |  |  |  |  |  |  |
| **TMT-B (s)** | **Stroop** |  | 35.70 ± 12.22 | 34.23 ± 12.93 |  | 63.94 ± 24.16 | 59.67 ± 22.14 |  |
|  | **Reading** |  | 34.05 ± 13.23 | 28.22 ± 13.63 |  | 67.79 ± 23.55 | 55.46 ± 16.93 |  |
|  |  |  |  |  |  |  |  |  |
| **TMT (B-A) (s)** | **Stroop** |  | 19.71 ± 11.23 | 19.43 ± 12.07 |  | 33.52 ± 17.99 | 32.51 ± 18.94 |  |
|  | **Reading** |  | 17.56 ± 12.03 | 14.16 ± 12.40 |  | 38.73 ± 16.55 | 28.35 ± 14.25 |  |
|  |  |  |  |  |  |  |  |  |

Data are presented as mean ± standard deviation.

Tab.S7. Single- and dual-task performance of the young and old participants before and after the interventions.

| **Task** | **Condition** |  | **YOUNG** | | | | |  | **OLD** | | | | |
| --- | --- | --- | --- | --- | --- | --- | --- | --- | --- | --- | --- | --- | --- |
|  |  |  |  | | | | |  |  | | | | |
|  |  |  | Single-Task | |  | Dual-Task | |  | Single-Task | |  | Dual-Task | |
|  |  |  |  | |  |  | |  |  | |  |  | |
|  |  |  | Pre | Post |  | Pre | Post |  | Pre | Post |  | Pre | Post |
| **Wordlist generation task** |  |  |  |  |  |  |  |  |  |  |  |  |  |
| (Number of words in 30s) | **Stroop** |  | 14.4 ± 4.0 | 16.8 ± 3.9 |  | 17.6 ± 3.9 | 17.4 ± 4.2 |  | 13.2 ± 3.1 | 13.9 ± 3.1 |  | 14.8 ± 3.5 | 15.1 ± 3.2 |
|  | **Reading** |  | 16.0 ± 3.8 | 16.7 ± 3.9 |  | 16.5 ± 4.7 | 18.2 ± 4.6 |  | 14.8 ± 2.8 | 14.1 ± 2.4 |  | 13.5 ± 2.6 | 14.5 ± 2.5 |
|  |  |  |  |  |  |  |  |  |  |  |  |  |  |
| **Arithmetic task** |  |  |  |  |  |  |  |  |  |  |  |  |  |
| (Number of correct responses in 30s) | **Stroop** |  | 19.7 ± 5.5 | 19.0 ± 4.9 |  | 20.6 ± 4.8 | 20.3 ± 5.6 |  | 16.3 ± 5.5 | 15.9 ± 6.0 |  | 16.9 ± 5.1 | 16.5 ± 5.4 |
|  | **Reading** |  | 20.2 ± 5.7 | 19.8 ± 5.4 |  | 20.9 ± 5.7 | 20.4 ± 5.4 |  | 15.5 ± 5.9 | 15.8 ± 5.8 |  | 17.1 ± 5.5 | 16.8 ± 5.9 |
|  |  |  |  |  |  |  |  |  |  |  |  |  |  |
| **Stroop task** |  |  |  |  |  |  |  |  |  |  |  |  |  |
| (Number of correct responses in 30s) | **Stroop** |  | 14.9 ± 0.3 | 14.9 ± 0.2 |  | 14.9 ± 0.2 | 14.9 ± 0.3 |  | 14.7 ± 0.6 | 14.6 ± 0.7 |  | 14.8 ± 0.5 | 14.7 ± 0.6 |
|  | **Reading** |  | 15.0 ± 0.1 | 14.9 ± 0.2 |  | 15.0 ± 0.1 | 15.0 ± 0.1 |  | 14.8 ± 0.3 | 14.9 ± 0.2 |  | 14.8 ± 0.3 | 14.8 ± 0.2 |
|  |  |  |  |  |  |  |  |  |  |  |  |  |  |
|  |  |  |  |  |  |  |  |  |  |  |  |  |  |

Data are presented as mean ± standard deviation.

Tab.S8. Psychological responses of the young and old participants before and after the interventions.

| **Parameter** | **Condition** |  | **YOUNG** | |  | **OLD** | |
| --- | --- | --- | --- | --- | --- | --- | --- |
|  |  |  |  | |  |  | |
|  |  |  | **Pre** | **Post** |  | **Pre** | **Post** |
|  |  |  |  |  |  |  |  |
| **POMS-F** | **Stroop** |  | 6.04 ± 4.36 | 12.38 ± 9.13 |  | 3.39 ± 5.31 | 4.96 ± 6.70 |
|  | **Reading** |  | 5.58 ± 4.91 | 7.88 ± 5.70 |  | 3.09 ± 4.66 | 4.57 ± 6.51 |
|  |  |  |  |  |  |  |  |
| **MDMQ** |  |  |  |  |  |  |  |
|  |  |  |  |  |  |  |  |
| Mood | **Stroop** |  | 17.08 ± 2.80 | 15.67 ± 1.93 |  | 18.00 ± 2.98 | 17.91 ± 2.52 |
| (positive-negative) | **Reading** |  | 17.71 ± 1.40 | 17.38 ± 1.41 |  | 18.48 ± 2.32 | 18.22 ± 2.69 |
|  |  |  |  |  |  |  |  |
| Wakefulness | **Stroop** |  | 14.58 ± 3.07 | 12.54 ± 3.40 |  | 17.13 ± 2.49 | 16.35 ± 2.91 |
| (awake-tired) | **Reading** |  | 15.04 ± 2.59 | 13.38 ± 3.20 |  | 17.22 ± 2.83 | 17.13 ± 2.61 |
|  |  |  |  |  |  |  |  |
| Arousal | **Stroop** |  | 15.50 ± 2.74 | 15.79 ± 2.57 |  | 17.61 ± 2.60 | 16.00 ± 2.59 |
| (calm-nervous) | **Reading** |  | 16.54 ± 2.52 | 17.54 ± 1.85 |  | 17.26 ± 3.38 | 16.78 ± 2.84 |
|  |  |  |  |  |  |  |  |
| **ASTS** |  |  |  |  |  |  |  |
|  |  |  |  |  |  |  |  |
| Mourning | **Stroop** |  | 4.38 ± 1.78 | 4.38 ± 2.12 |  | 3.61 ± 2.10 | 3.26 ± 1.22 |
|  | **Reading** |  | 3.88 ± 1.17 | 3.58 ± 1.11 |  | 3.78 ± 2.32 | 3.74 ± 2.00 |
|  |  |  |  |  |  |  |  |
| Hopelessness | **Stroop** |  | 3.50 ± 1.04 | 3.67 ± 1.37 |  | 3.83 ± 2.20 | 3.39 ± 1.84 |
|  | **Reading** |  | 3.21 ± 0.50 | 3.29 ± 0.54 |  | 3.65 ± 1.97 | 3.96 ± 2.48 |
|  |  |  |  |  |  |  |  |
| Tiredness | **Stroop** |  | 7.71 ± 2.95 | 10.79 ± 4.07 |  | 5.78 ± 3.18 | 6.65 ± 3.61 |
|  | **Reading** |  | 6.96 ± 3.12 | 8.92 ± 3.86 |  | 6.30 ± 3.20 | 6.22 ± 3.05 |
|  |  |  |  |  |  |  |  |
| Positive Mood | **Stroop** |  | 28.50 ± 5.40 | 23.88 ± 7.61 |  | 30.30 ± 6.45 | 30.04 ± 8.05 |
|  | **Reading** |  | 29.08 ± 5.55 | 26.79 ± 6.29 |  | 29.48 ± 7.86 | 30.43 ± 6.35 |
|  |  |  |  |  |  |  |  |
| Anger | **Stroop** |  | 3.38 ± 0.63 | 4.38 ± 2.41 |  | 3.13 ± 0.45 | 3.35 ± 1.27 |
|  | **Reading** |  | 3.04 ± 0.20 | 3.00 ± 0.00 |  | 3.04 ± 0.20 | 3.22 ± 0.83 |
|  |  |  |  |  |  |  |  |

POMS-F. Profile of Mood States-Fatigue; MDMQ. Multidimensional Mood Questionnaire; ASTS. Aktuelle Stimmungsskala (German Current Mood Scale), Date are presented as mean ± standard deviation.

Tab.S9. Retrospective rating of stress and attention/ prioritization via visual analog scales according to the dual-task walking of young and old participants before and after the interventions.

| **Parameter** | **Condition** |  |  | | | | | | | |
| --- | --- | --- | --- | --- | --- | --- | --- | --- | --- | --- |
|  |  |  |  | |  | ***YOUNG*** | |  |  | |
|  |  |  |  | |  |  | |  |  | |
|  |  |  | **WLG** | |  | **AT** | |  | **STR** | |
|  |  |  | **Pre** | **Post** |  | **Pre** | **Post** |  | **Pre** | **Post** |
| **Visual Analog Scale** |  |  |  |  |  |  |  |  |  |  |
|  |  |  |  |  |  |  |  |  |  |  |
| Stress | **Stroop** |  | 38.5 ± 22.1 | 40.4 ± 20.1 |  | 36.5 ± 17.7 | 37.8 ± 22.7 |  | 31.8 ± 19.0 | 34.6 ± 17.9 |
|  | **Reading** |  | 36.9 ± 18.1 | 32.2 ± 21.4 |  | 38.4 ± 19.2 | 33.8 ± 19.7 |  | 28.0 ± 18.4 | 24.8 ± 19.0 |
|  |  |  |  | | | | | | | |
|  |  |  |  |  |  | ***OLD*** | |  |  |  |
|  |  |  |  |  |  |  |  |  |  |  |
|  | **Stroop** |  | 35.7 ± 27.5 | 28.9 ± 25.9 |  | 32.0 ± 25.4 | 26.0 ± 22.9 |  | 20.1 ± 20.8 | 20.1 ± 21.5 |
|  | **Reading** |  | 30.7 ± 21.6 | 28.8 ± 22.3 |  | 35.2 ± 26.5 | 34.5 ± 24.5 |  | 26.0 ± 22.2 | 21.7 ± 20.4 |
|  |  |  |  |  |  |  |  |  |  |  |
|  |  |  |  |  |  |  |  |  |  |  |
|  |  |  | ***YOUNG*** | | | | | | | |
|  |  |  |  | |  |  | |  |  | |
|  |  |  | **WLG** | |  | **AT** | |  | **STR** | |
| **Visual Analog Scale** |  |  | **Pre** | **Post** |  | **Pre** | **Post** |  | **Pre** | **Post** |
|  |  |  |  |  |  |  |  |  |  |  |
| Prioritization/ Attention |  |  |  |  |  |  |  |  |  |  |
| (motor task – cognitive task) | **Stroop** |  | 75.9 ± 12.9 | 73.7 ± 14.6 |  | 75.5 ± 12.9 | 72.2 ± 13.1 |  | 69.9 ± 15.1 | 70.1 ± 11.9 |
|  | **Reading** |  | 76.8 ± 12.9 | 75.3 ± 14.8 |  | 73.5 ± 17.2 | 73.9 ± 13.4 |  | 72.5 ± 16.7 | 70.9 ± 15.5 |
|  |  |  |  |  |  |  |  |  |  |  |
|  |  |  | ***OLD*** | | | | | | | |
|  |  |  |  |  |  |  |  |  |  |  |
|  | **Stroop** |  | 76.0 ± 17.8 | 75.7 ± 16.8 |  | 73.7 ± 18.7 | 75.3 ± 19.8 |  | 73.0 ± 17.4 | 72.5 ± 20.4 |
|  | **Reading** |  | 72.5 ± 18.7 | 70.0 ± 22.5 |  | 72.7 ± 20.6 | 71.5 ± 25.5 |  | 72.0 ± 18.7 | 67.3 ± 25.6 |
|  |  |  |  |  |  |  |  |  |  |  |
|  |  |  |  |  |  |  |  |  |  |  |

ST, Single-Task; WLG, Word list generation task; AT, Arithmetic task; STR, Stroop-task; Data are presented as mean ± standard deviation.

Tab.S10. Statistical outcomes (ANOVA).

|  |  | | |  | | |  | | |  | | |  | | |  | | |  | | |
| --- | --- | --- | --- | --- | --- | --- | --- | --- | --- | --- | --- | --- | --- | --- | --- | --- | --- | --- | --- | --- | --- |
|  | **Condition** | | | **Time** | | | **Group** | | | **Condition x Time** | | | **Condition x Group** | | | **Time x Group** | | | **Condition x Time x Group** | | |
|  | **F** | ***P*** | **Ƞ_p_^2^** | **F** | ***P*** | **Ƞ_p_^2^** | **F** | ***P*** | **Ƞ_p_^2^** | **F** | ***P*** | **Ƞ_p_^2^** | **F** | ***P*** | **Ƞ_p_^2^** | **F** | ***P*** | **Ƞ_p_^2^** | **F** | ***P*** | **Ƞ_p_^2^** |
|  |  |  |  |  |  |  |  |  |  |  |  |  |  |  |  |  |  |  |  |  |  |
| POMS-F | 3.405 | **0.072^†^** | 0.072 | 8.906 | **0.005**** | 0.168 | 6.985 | **0.011*** | 0.137 | 2.704 | 0.107 | 0.058 | 2.010 | 0.163 | 0.044 | 2.578 | 0.116 | 0.055 | 1.788 | 0.188 | 0.039 |
|  |  |  |  |  |  |  |  |  |  |  |  |  |  |  |  |  |  |  |  |  |  |
| MDMQ |  |  |  |  |  |  |  |  |  |  |  |  |  |  |  |  |  |  |  |  |  |
| Mood  (positive-negative) | 1.994 | 0.165 | 0.043 | 1.021 | 0.318 | 0.023 | 4.810 | **0.034*** | 0.099 | 0.000 | 0.989 | 0.000 | 2.046 | 0.160 | 0.044 | 2.297 | 0.137 | 0.050 | 3.474 | **0.069^†^** | 0.073 |
| Wakefulness  (awake-tired) | 1.262 | 0.267 | 0.028 | 10.136 | **0.003**** | 0.187 | 17.427 | **0.000**** | 0.284 | 0.612 | 0.438 | 0.014 | 0.035 | 0.853 | 0.001 | 3.382 | **0.073^†^** | 0.071 | 0.183 | 0.671 | 0.004 |
| Arousal  (calm-nervous) | 1.610 | 0.211 | 0.035 | 0.145 | 0.705 | 0.003 | 0.977 | 0.328 | 0.022 | 2.495 | 0.121 | 0.054 | 0.413 | 0.524 | 0.009 | 2.320 | 0.135 | 0.050 | 0.549 | 0.462 | 0.012 |
|  |  |  |  |  |  |  |  |  |  |  |  |  |  |  |  |  |  |  |  |  |  |
| ASTS |  |  |  |  |  |  |  |  |  |  |  |  |  |  |  |  |  |  |  |  |  |
| Mourning | 2.449 | 0.125 | 0.053 | 0.315 | 0.578 | 0.007 | 1.319 | 0.257 | 0.029 | 0.686 | 0.412 | 0.015 | 5.538 | **0.023*** | 0.112 | 0.006 | 0.937 | 0.000 | 0.763 | 0.387 | 0.017 |
| Hopelessness | 0.024 | 0.878 | 0.001 | 0.169 | 0.683 | 0.004 | 0.169 | 0.683 | 0.004 | 0.099 | 0.754 | 0.002 | 2.142 | 0.150 | 0.046 | 0.459 | 0.502 | 0.010 | 1.043 | 0.313 | 0.023 |
| Tiredness | 1.980 | 0.166 | 0.043 | 3.151 | **0.083^†^** | 0.067 | 8.408 | **0.006**** | 0.160 | 0.482 | 0.491 | 0.011 | 3.162 | **0.082^†^** | 0.067 | 9.639 | **0.003**** | 0.180 | 0.024 | 0.878 | 0.001 |
| Positive Mood | 1.069 | 0.307 | 0.024 | 2.344 | 0.133 | 0.051 | 4.027 | **0.051^†^** | 0.084 | 0.000 | 0.998 | 0.000 | 1.312 | 0.258 | 0.029 | 8.523 | **0.006**** | 0.162 | 0.407 | 0.527 | 0.009 |
| Anger | 1.554 | 0.219 | 0.034 | 1.734 | 0.195 | 0.038 | 1.750 | 0.193 | 0.038 | 0.967 | 0.331 | 0.022 | 4.548 | **0.039*** | 0.094 | 0.526 | 0.472 | 0.012 | 2.343 | 0.133 | 0.051 |
|  |  |  |  |  |  |  |  |  |  |  |  |  |  |  |  |  |  |  |  |  |  |
| Gait Parameter |  |  |  |  |  |  |  |  |  |  |  |  |  |  |  |  |  |  |  |  |  |
|  |  |  |  |  |  |  |  |  |  |  |  |  |  |  |  |  |  |  |  |  |  |
| *Single-Task* |  |  |  |  |  |  |  |  |  |  |  |  |  |  |  |  |  |  |  |  |  |
|  |  |  |  |  |  |  |  |  |  |  |  |  |  |  |  |  |  |  |  |  |  |
| *Step width (m)* | 3.863 | **0.056^†^** | 0.081 | 6.417 | **0.015*** | 0.127 | 0.753 | 0.390 | 0.017 | 2.581 | 0.115 | 0.055 | 0.008 | 0.931 | 0.000 | 0.866 | 0.357 | 0.019 | 1.857 | 0.180 | 0.040 |
| *Step length (m)* | 0.411 | 0.525 | 0.009 | 6.932 | **0.012*** | 0.136 | 58.219 | **0.000**** | 0.570 | 3.494 | **0.068^†^** | 0.074 | 0.137 | 0.713 | 0.003 | 6.033 | **0.018*** | 0.121 | 1.312 | 0.258 | 0.029 |
| *Stride length (m)* | 1.512 | 0.225 | 0.033 | 1.902 | 0.175 | 0.041 | 64.293 | **0.000**** | 0.594 | 1.871 | 0.178 | 0.041 | 0.240 | 0.627 | 0.005 | 5.452 | **0.024*** | 0.110 | 0.059 | 0.810 | 0.001 |
| *Double Support time (s)* | 0.434 | 0.513 | 0.010 | 0.641 | 0.428 | 0.014 | 15.961 | **0.000**** | 0.266 | 0.634 | 0.430 | 0.014 | 0.634 | 0.430 | 0.014 | 0.443 | 0.509 | 0.010 | 0.005 | 0.943 | 0.000 |
| *Stride duration (s)* | 1.766 | 0.191 | 0.039 | 1.756 | 0.192 | 0.038 | 3.564 | **0.066^†^** | 0.075 | 0.556 | 0.460 | 0.012 | 0.349 | 0.558 | 0.008 | 5.085 | **0.029*** | 0.104 | 0.427 | 0.517 | 0.010 |
| *Stand duration (s)* | 1.365 | 0.249 | 0.030 | 1.382 | 0.246 | 0.030 | 7.418 | **0.009**** | 0.144 | 0.691 | 0.410 | 0.015 | 0.073 | 0.788 | 0.002 | 3.364 | **0.073^†^** | 0.071 | 0.285 | 0.596 | 0.006 |
| *Swing duration (s)* | 2.117 | 0.153 | 0.046 | 2.073 | 0.157 | 0.045 | 0.018 | 0.893 | 0.000 | 0.420 | 0.520 | 0.009 | 1.514 | 0.225 | 0.033 | 7.891 | **0.007**** | 0.152 | 0.845 | 0.363 | 0.019 |
| *Stand phase (%)* | 0.091 | 0.765 | 0.002 | 0.093 | 0.762 | 0.002 | 20.027 | **0.000**** | 0.313 | 0.754 | 0.390 | 0.017 | 2.455 | 0.124 | 0.053 | 0.239 | 0.627 | 0.005 | 0.245 | 0.623 | 0.006 |
| *Swing phase (%)* | 0.066 | 0.798 | 0.002 | 0.079 | 0.780 | 0.002 | 19.867 | **0.000**** | 0.311 | 0.284 | 0.597 | 0.006 | 2.332 | 0.134 | 0.050 | 0.329 | 0.569 | 0.007 | 0.281 | 0.599 | 0.006 |
| *Cadence (steps/ min)* | 1.547 | 0.220 | 0.034 | 1.290 | 0.262 | 0.028 | 3.079 | **0.086^†^** | 0.065 | 0.408 | 0.527 | 0.009 | 0.303 | 0.585 | 0.007 | 4.693 | **0.036*** | 0.096 | 0.378 | 0.542 | 0.009 |
|  |  |  |  |  |  |  |  |  |  |  |  |  |  |  |  |  |  |  |  |  |  |
| *CV _Step width_ (%)* | 2.209 | 0.144 | 0.048 | 2.393 | 0.129 | 0.052 | 1.037 | 0.314 | 0.023 | 1.317 | 0.257 | 0.029 | 2.435 | 0.126 | 0.052 | 0.38 | 0.541 | 0.009 | 0.748 | 0.392 | 0.017 |
| *CV _Step length_ (%)* | 0.483 | 0.491 | 0.011 | 0.001 | 0.980 | 0.000 | 87.904 | **0.000**** | 0.666 | 0.024 | 0.876 | 0.001 | 0.011 | 0.915 | 0.000 | 0.111 | 0.741 | 0.003 | 2.995 | **0.091^†^** | 0.064 |
| *CV _Stride length_ (%)* | 1.847 | 0.181 | 0.040 | 0.330 | 0.569 | 0.007 | 103.175 | **0.000**** | 0.701 | 0.143 | 0.707 | 0.003 | 0.240 | 0.627 | 0.005 | 0.042 | 0.839 | 0.001 | 2.023 | 0.162 | 0.044 |
| *CV _Double support time_ (%)* | 0.846 | 0.363 | 0.019 | 0.921 | 0.342 | 0.021 | 26.656 | **0.000**** | 0.377 | 0.514 | 0.477 | 0.012 | 1.167 | 0.286 | 0.026 | 0.919 | 0.343 | 0.020 | 0.059 | 0.809 | 0.001 |
| *CV _Stride duration_ (%)* | 0.001 | 0.979 | 0.000 | 0.009 | 0.923 | 0.000 | 60.521 | **0.000**** | 0.579 | 0.102 | 0.751 | 0.002 | 1.301 | 0.260 | 0.029 | 0.222 | 0.640 | 0.005 | 0.373 | 0.545 | 0.008 |
| *CV _Stand duration_ (%)* | 1.115 | 0.297 | 0.025 | 0.092 | 0.763 | 0.002 | 55.657 | **0.000**** | 0.558 | 0.015 | 0.903 | 0.000 | 0.240 | 0.626 | 0.005 | 1.234 | 0.273 | 0.027 | 0.213 | 0.647 | 0.005 |
| *CV _Swing duration_ (%)* | 0.904 | 0.347 | 0.020 | 0.291 | 0.592 | 0.007 | 69.390 | **0.000**** | 0.612 | 1.725 | 0.196 | 0.038 | 0.177 | 0.676 | 0.004 | 0.000 | 0.994 | 0.000 | 2.916 | **0.095^†^** | 0.062 |
| *CV _Stand phase_ (%)* | 0.175 | 0.678 | 0.004 | 0.544 | 0.465 | 0.012 | 47.695 | **0.000**** | 0.520 | 1.066 | 0.307 | 0.024 | 0.037 | 0.848 | 0.001 | 0.152 | 0.698 | 0.003 | 0.592 | 0.446 | 0.013 |
| *CV _Swing phase_ (%)* | 0.011 | 0.916 | 0.000 | 0.446 | 0.508 | 0.010 | 50.869 | **0.000**** | 0.536 | 1.242 | 0.271 | 0.027 | 0.077 | 0.782 | 0.002 | 0.010 | 0.920 | 0.000 | 0.129 | 0.721 | 0.003 |
| *CV _Cadence_ (%)* | 0.548 | 0.463 | 0.012 | 0.123 | 0.727 | 0.003 | 62.695 | **0.000**** | 0.588 | 0.033 | 0.858 | 0.001 | 0.825 | 0.369 | 0.018 | 0.379 | 0.541 | 0.009 | 0.218 | 0.643 | 0.005 |
|  |  |  |  |  |  |  |  |  |  |  |  |  |  |  |  |  |  |  |  |  |  |
| Dual-Task |  |  |  |  |  |  |  |  |  |  |  |  |  |  |  |  |  |  |  |  |  |
| *WLG* |  |  |  |  |  |  |  |  |  |  |  |  |  |  |  |  |  |  |  |  |  |
|  |  |  |  |  |  |  |  |  |  |  |  |  |  |  |  |  |  |  |  |  |  |
| *Step width (m)* | 0.530 | 0.471 | 0.012 | 3.130 | **0.084^†^** | 0.066 | 2.063 | 0.158 | 0.045 | 1.944 | 0.170 | 0.042 | 0.060 | 0.807 | 0.001 | 0.082 | 0.776 | 0.002 | 0.043 | 0.837 | 0.001 |
| *Step length (m)* | 0.007 | 0.933 | 0.000 | 1.190 | 0.281 | 0.026 | 54.325 | **0.000**** | 0.553 | 0.303 | 0.585 | 0.007 | 0.635 | 0.430 | 0.014 | 0.569 | 0.455 | 0.013 | 1.506 | 0.226 | 0.033 |
| *Stride length (m)* | 0.008 | 0.928 | 0.000 | 0.198 | 0.659 | 0.004 | 58.631 | **0.000**** | 0.571 | 0.021 | 0.886 | 0.000 | 0.528 | 0.471 | 0.012 | 1.208 | 0.278 | 0.027 | 0.184 | 0.670 | 0.004 |
| *Double support time (s)* | 0.000 | 0.991 | 0.000 | 2.838 | **0.099^†^** | 0.061 | 13.734 | **0.001**** | 0.238 | 0.366 | 0.548 | 0.008 | 0.029 | 0.865 | 0.001 | 0.154 | 0.696 | 0.003 | 1.243 | 0.271 | 0.027 |
| *Stride duration (s)* | 0.013 | 0.909 | 0.000 | 0.699 | 0.408 | 0.016 | 4.132 | **0.048*** | 0.086 | 0.146 | 0.704 | 0.003 | 0.236 | 0.629 | 0.005 | 1.425 | 0.239 | 0.031 | 0.884 | 0.352 | 0.020 |
| *Stand duration (s)* | 0.008 | 0.929 | 0.000 | 1.394 | 0.244 | 0.031 | 7.193 | **0.010**** | 0.141 | 0.304 | 0.584 | 0.007 | 0.132 | 0.718 | 0.003 | 1.046 | 0.312 | 0.023 | 1.268 | 0.266 | 0.028 |
| *Swing duration (s)* | 0.047 | 0.830 | 0.001 | 0.010 | 0.922 | 0.000 | 0.113 | 0.738 | 0.003 | 0.005 | 0.943 | 0.000 | 0.265 | 0.609 | 0.006 | 1.618 | 0.210 | 0.035 | 0.115 | 0.736 | 0.003 |
| *Stand phase (%)* | 0.028 | 0.867 | 0.001 | 3.078 | **0.086^†^** | 0.065 | 17.326 | **0.000**** | 0.283 | 0.383 | 0.539 | 0.009 | 0.058 | 0.810 | 0.001 | 0.187 | 0.667 | 0.004 | 0.794 | 0.378 | 0.018 |
| *Swing phase (%)* | 0.028 | 0.867 | 0.001 | 2.803 | 0.101 | 0.060 | 17.260 | **0.000**** | 0.282 | 0.372 | 0.545 | 0.008 | 0.037 | 0.848 | 0.001 | 0.226 | 0.637 | 0.005 | 0.831 | 0.367 | 0.019 |
| *Cadence (steps/ min)* | 0.000 | 0.994 | 0.000 | 0.254 | 0.617 | 0.006 | 3.437 | **0.070^†^** | 0.072 | 0.052 | 0.820 | 0.001 | 0.194 | 0.662 | 0.004 | 1.059 | 0.309 | 0.023 | 0.825 | 0.369 | 0.018 |
|  |  |  |  |  |  |  |  |  |  |  |  |  |  |  |  |  |  |  |  |  |  |
| *CV _Step width_ (%)* | 0.262 | 0.611 | 0.006 | 4.490 | **0.040*** | 0.093 | 0.126 | 0.724 | 0.003 | 3.311 | **0.076^†^** | 0.070 | 0.013 | 0.910 | 0.000 | 0.328 | 0.570 | 0.007 | 0.023 | 0.880 | 0.001 |
| *CV _Step length_ (%)* | 0.591 | 0.446 | 0.013 | 0.409 | 0.526 | 0.009 | 0.051 | 0.823 | 0.001 | 0.006 | 0.937 | 0.000 | 0.409 | 0.526 | 0.009 | 0.104 | 0.749 | 0.002 | 0.051 | 0.823 | 0.001 |
| *CV _Stride length_ (%)* | 0.569 | 0.455 | 0.013 | 1.176 | 0.284 | 0.026 | 59.737 | **0.000**** | 0.576 | 0.071 | 0.791 | 0.002 | 0.317 | 0.576 | 0.007 | 1.670 | 0.203 | 0.037 | 0.002 | 0.968 | 0.000 |
| *CV _Double support time_ (%)* | 5.408 | 0.025 | 0.112 | 0.581 | 0.450 | 0.013 | 33.962 | **0.000**** | 0.441 | 0.138 | 0.712 | 0.003 | 14.813 | **0.000**** | 0.256 | 0.165 | 0.687 | 0.004 | 0.614 | 0.438 | 0.014 |
| *CV _Stride duration_ (%)* | 0.015 | 0.903 | 0.000 | 0.012 | 0.914 | 0.000 | 53.894 | **0.000**** | 0.551 | 1.315 | 0.258 | 0.029 | 0.807 | 0.374 | 0.018 | 3.256 | **0.078^†^** | 0.069 | 0.000 | 0.995 | 0.000 |
| *CV _Stand duration_ (%)* | 0.190 | 0.665 | 0.004 | 0.323 | 0.573 | 0.007 | 52.116 | **0.000**** | 0.542 | 0.804 | 0.375 | 0.018 | 0.214 | 0.646 | 0.005 | 0.312 | 0.579 | 0.007 | 0.160 | 0.691 | 0.004 |
| *CV _Swing duration_ (%)* | 0.049 | 0.825 | 0.001 | 0.017 | 0.898 | 0.000 | 90.971 | **0.000**** | 0.674 | 0.176 | 0.677 | 0.004 | 0.742 | 0.394 | 0.017 | 0.240 | 0.626 | 0.005 | 0.402 | 0.529 | 0.009 |
| *CV _Stand phase_ (%)* | 0.004 | 0.953 | 0.000 | 0.205 | 0.653 | 0.005 | 77.015 | **0.000**** | 0.636 | 0.014 | 0.906 | 0.000 | 0.197 | 0.659 | 0.004 | 0.399 | 0.531 | 0.009 | 1.414 | 0.241 | 0.031 |
| *CV _Swing phase_ (%)* | 0.011 | 0.916 | 0.000 | 0.052 | 0.820 | 0.001 | 85.967 | **0.000**** | 0.661 | 0.001 | 0.972 | 0.000 | 0.213 | 0.646 | 0.005 | 0.750 | 0.391 | 0.017 | 1.982 | 0.166 | 0.043 |
| *CV _Cadence_ (%)* | 0.000 | 0.995 | 0.000 | 0.069 | 0.793 | 0.002 | 53.225 | **0.000**** | 0.547 | 1.977 | 0.167 | 0.043 | 0.176 | 0.677 | 0.004 | 2.234 | 0.142 | 0.048 | 0.012 | 0.913 | 0.000 |
|  |  |  |  |  |  |  |  |  |  |  |  |  |  |  |  |  |  |  |  |  |  |
| Dual-Task |  |  |  |  |  |  |  |  |  |  |  |  |  |  |  |  |  |  |  |  |  |
| *Arithmetic task* |  |  |  |  |  |  |  |  |  |  |  |  |  |  |  |  |  |  |  |  |  |
|  |  |  |  |  |  |  |  |  |  |  |  |  |  |  |  |  |  |  |  |  |  |
| *Step width (m)* | 1.062 | 0.308 | 0.024 | 13.841 | **0.001**** | 0.239 | 1.563 | 0.218 | 0.034 | 0.165 | 0.687 | 0.004 | 0.001 | 0.980 | 0.000 | 0.691 | 0.410 | 0.015 | 0.811 | 0.373 | 0.018 |
| *Step length (m)* | 0.592 | 0.446 | 0.013 | 0.335 | 0.566 | 0.008 | 58.215 | **0.000**** | 0.570 | 0.515 | 0.477 | 0.012 | 3.707 | **0.061^†^** | 0.078 | 2.339 | 0.133 | 0.050 | 0.626 | 0.433 | 0.014 |
| *Stride length (m)* | 1.241 | 0.271 | 0.027 | 0.324 | 0.572 | 0.007 | 65.135 | **0.000**** | 0.597 | 0.181 | 0.672 | 0.004 | 2.641 | 0.111 | 0.057 | 1.699 | 0.199 | 0.037 | 0.066 | 0.799 | 0.001 |
| *Double support time (s)* | 0.006 | 0.940 | 0.000 | 0.024 | 0.877 | 0.001 | 12.366 | **0.001**** | 0.219 | 0.893 | 0.350 | 0.020 | 2.281 | 0.138 | 0.049 | 2.615 | 0.113 | 0.056 | 0.170 | 0.682 | 0.004 |
| *Stride duration (s)* | 0.909 | 0.346 | 0.020 | 0.274 | 0.603 | 0.006 | 2.871 | **0.097^†^** | 0.061 | 0.661 | 0.421 | 0.015 | 2.020 | 0.162 | 0.044 | 2.187 | 0.146 | 0.047 | 0.414 | 0.523 | 0.009 |
| *Stand duration (s)* | 0.504 | 0.481 | 0.011 | 0.172 | 0.680 | 0.004 | 5.702 | **0.021*** | 0.115 | 0.917 | 0.344 | 0.020 | 2.274 | 0.139 | 0.049 | 2.468 | 0.123 | 0.053 | 0.171 | 0.681 | 0.004 |
| *Swing duration (s)* | 1.846 | 0.181 | 0.040 | 0.645 | 0.426 | 0.014 | 0.015 | 0.904 | 0.000 | 0.479 | 0.493 | 0.011 | 1.203 | 0.279 | 0.027 | 1.656 | 0.205 | 0.036 | 0.378 | 0.542 | 0.009 |
| *Stand phase (%)* | 0.536 | 0.468 | 0.012 | 0.249 | 0.620 | 0.006 | 17.178 | **0.000**** | 0.281 | 1.005 | 0.322 | 0.022 | 1.243 | 0.271 | 0.027 | 2.938 | **0.094^†^** | 0.063 | 0.014 | 0.907 | 0.000 |
| *Swing phase (%)* | 0.494 | 0.486 | 0.011 | 0.553 | 0.461 | 0.012 | 16.890 | **0.000**** | 0.277 | 0.848 | 0.362 | 0.019 | 1.194 | 0.280 | 0.026 | 2.084 | 0.156 | 0.045 | 0.008 | 0.928 | 0.000 |
| *Cadence (steps/ min)* | 1.013 | 0.320 | 0.022 | 0.150 | 0.701 | 0.003 | 2.269 | 0.139 | 0.049 | 0.867 | 0.357 | 0.019 | 2.019 | 0.162 | 0.044 | 2.120 | 0.153 | 0.046 | 0.417 | 0.522 | 0.009 |
|  |  |  |  |  |  |  |  |  |  |  |  |  |  |  |  |  |  |  |  |  |  |
| *CV _Step width_ (%)* | 0.579 | 0.451 | 0.013 | 12.031 | **0.001**** | 0.215 | 0.064 | 0.801 | 0.001 | 2.271 | 0.139 | 0.049 | 0.287 | 0.595 | 0.006 | 0.001 | 0.971 | 0.000 | 2.998 | **0.090^†^** | 0.064 |
| *CV _Step length_ (%)* | 0.970 | 0.330 | 0.022 | 0.499 | 0.484 | 0.011 | 75.346 | **0.000**** | 0.631 | 0.758 | 0.389 | 0.017 | 0.036 | 0.851 | 0.001 | 3.144 | **0.083^†^** | 0.067 | 0.241 | 0.626 | 0.005 |
| *CV _Stride length_ (%)* | 0.578 | 0.451 | 0.013 | 0.263 | 0.610 | 0.006 | 70.688 | **0.000**** | 0.616 | 0.340 | 0.563 | 0.008 | 0.006 | 0.939 | 0.000 | 2.003 | 0.164 | 0.044 | 0.310 | 0.580 | 0.007 |
| *CV _Double support time_ (%)* | 0.124 | 0.727 | 0.003 | 0.853 | 0.361 | 0.019 | 58.313 | **0.000**** | 0.570 | 1.304 | 0.260 | 0.029 | 0.111 | 0.741 | 0.003 | 0.765 | 0.387 | 0.017 | 0.007 | 0.934 | 0.000 |
| *CV _Stride duration_ (%)* | 0.070 | 0.793 | 0.002 | 3.111 | **0.085^†^** | 0.066 | 61.585 | **0.000**** | 0.583 | 1.693 | 0.200 | 0.037 | 0.128 | 0.723 | 0.003 | 0.908 | 0.346 | 0.020 | 1.091 | 0.302 | 0.024 |
| *CV _Stand duration_ (%)* | 0.095 | 0.759 | 0.002 | 2.852 | **0.098^†^** | 0.061 | 76.794 | **0.000**** | 0.636 | 0.286 | 0.595 | 0.006 | 0.228 | 0.636 | 0.005 | 0.670 | 0.418 | 0.015 | 0.114 | 0.737 | 0.003 |
| *CV _Swing duration_ (%)* | 2.179 | 0.147 | 0.047 | 3.067 | **0.087^†^** | 0.065 | 96.776 | **0.000**** | 0.687 | 2.218 | 0.144 | 0.048 | 0.437 | 0.512 | 0.010 | 1.809 | 0.186 | 0.039 | 0.007 | 0.934 | 0.000 |
| *CV _Stand phase_ (%)* | 3.138 | **0.083^†^** | 0.067 | 1.096 | 0.301 | 0.024 | 92.343 | **0.000**** | 0.677 | 0.039 | 0.844 | 0.001 | 0.321 | 0.574 | 0.007 | 0.166 | 0.685 | 0.004 | 0.407 | 0.527 | 0.009 |
| *CV _Swing phase_ (%)* | 3.064 | **0.087^†^** | 0.065 | 1.437 | 0.237 | 0.032 | 92.324 | **0.000**** | 0.677 | 0.010 | 0.922 | 0.000 | 0.149 | 0.701 | 0.003 | 0.180 | 0.673 | 0.004 | 0.266 | 0.608 | 0.006 |
| *CV _Cadence_ (%)* | 0.183 | 0.671 | 0.004 | 1.725 | 0.196 | 0.038 | 55.549 | **0.000**** | 0.558 | 1.735 | 0.195 | 0.038 | 0.173 | 0.679 | 0.004 | 0.154 | 0.697 | 0.003 | 0.442 | 0.510 | 0.010 |
|  |  |  |  |  |  |  |  |  |  |  |  |  |  |  |  |  |  |  |  |  |  |
| Dual-Task |  |  |  |  |  |  |  |  |  |  |  |  |  |  |  |  |  |  |  |  |  |
| *Stroop-Task* |  |  |  |  |  |  |  |  |  |  |  |  |  |  |  |  |  |  |  |  |  |
|  |  |  |  |  |  |  |  |  |  |  |  |  |  |  |  |  |  |  |  |  |  |
| *Step width (m)* | 3.722 | **0.060^†^** | 0.078 | 9.295 | **0.004**** | 0.174 | 2.284 | 0.138 | 0.049 | 0.021 | 0.885 | 0.000 | 0.037 | 0.847 | 0.001 | 1.617 | 0.210 | 0.035 | 0.785 | 0.380 | 0.018 |
| *Step length (m)* | 0.307 | 0.582 | 0.007 | 0.121 | 0.730 | 0.003 | 63.377 | **0.000**** | 0.590 | 0.000 | 0.988 | 0.000 | 1.282 | 0.264 | 0.028 | 1.224 | 0.275 | 0.027 | 4.021 | **0.051^†^** | 0.084 |
| *Stride length (m)* | 0.591 | 0.446 | 0.013 | 0.054 | 0.818 | 0.001 | 70.007 | **0.000**** | 0.614 | 0.110 | 0.742 | 0.002 | 0.774 | 0.384 | 0.017 | 1.019 | 0.318 | 0.023 | 3.098 | **0.085^†^** | 0.066 |
| *Double support time (s)* | 0.094 | 0.761 | 0.002 | 0.276 | 0.602 | 0.006 | 11.101 | **0.002**** | 0.201 | 0.310 | 0.581 | 0.007 | 0.870 | 0.356 | 0.019 | 2.481 | 0.122 | 0.053 | 3.189 | **0.081^†^** | 0.068 |
| *Stride duration (s)* | 0.687 | 0.412 | 0.015 | 0.012 | 0.912 | 0.000 | 1.329 | 0.255 | 0.029 | 0.011 | 0.917 | 0.000 | 0.519 | 0.475 | 0.012 | 1.438 | 0.237 | 0.032 | 3.537 | **0.067^†^** | 0.074 |
| *Stand duration (s)* | 0.480 | 0.492 | 0.011 | 0.064 | 0.801 | 0.001 | 3.775 | **0.058^†^** | 0.079 | 0.005 | 0.947 | 0.000 | 0.611 | 0.439 | 0.014 | 1.878 | 0.178 | 0.041 | 3.762 | **0.059^†^** | 0.079 |
| *Swing duration (s)* | 1.117 | 0.296 | 0.025 | 0.007 | 0.934 | 0.000 | 0.536 | 0.468 | 0.012 | 0.249 | 0.620 | 0.006 | 0.276 | 0.602 | 0.006 | 0.998 | 0.323 | 0.022 | 2.912 | **0.095^†^** | 0.062 |
| *Stand phase (%)* | 0.046 | 0.832 | 0.001 | 0.614 | 0.437 | 0.014 | 17.552 | **0.000**** | 0.285 | 1.415 | 0.241 | 0.031 | 0.525 | 0.472 | 0.012 | 2.335 | 0.134 | 0.050 | 2.536 | 0.118 | 0.054 |
| *Swing phase (%)* | 0.069 | 0.794 | 0.002 | 0.586 | 0.448 | 0.013 | 17.483 | **0.000**** | 0.284 | 1.039 | 0.314 | 0.023 | 0.572 | 0.454 | 0.013 | 2.271 | 0.139 | 0.049 | 2.923 | **0.094^†^** | 0.062 |
| *Cadence (steps/ min)* | 0.800 | 0.376 | 0.018 | 0.010 | 0.921 | 0.000 | 0.855 | 0.360 | 0.019 | 0.003 | 0.954 | 0.000 | 0.496 | 0.485 | 0.011 | 1.913 | 0.174 | 0.042 | 2.941 | **0.093^†^** | 0.063 |
|  |  |  |  |  |  |  |  |  |  |  |  |  |  |  |  |  |  |  |  |  |  |
| *CV _Step width_ (%)* | 1.966 | 0.168 | 0.043 | 5.590 | **0.023*** | 0.113 | 0.431 | 0.515 | 0.010 | 0.019 | 0.890 | 0.000 | 3.810 | **0.057^†^** | 0.080 | 0.232 | 0.632 | 0.005 | 0.159 | 0.692 | 0.004 |
| *CV _Step length_ (%)* | 1.996 | 0.165 | 0.043 | 0.015 | 0.902 | 0.000 | 59.157 | **0.000**** | 0.573 | 0.009 | 0.925 | 0.000 | 3.184 | **0.081^†^** | 0.067 | 5.839 | **0.020*** | 0.117 | 0.407 | 0.527 | 0.009 |
| *CV _Stride length_ (%)* | 0.988 | 0.326 | 0.022 | 0.317 | 0.576 | 0.007 | 56.103 | **0.000**** | 0.560 | 0.043 | 0.836 | 0.001 | 1.303 | 0.260 | 0.029 | 6.379 | **0.015*** | 0.127 | 0.994 | 0.324 | 0.022 |
| *CV _Double support time_ (%)* | 0.290 | 0.593 | 0.007 | 3.804 | 0.058 | 0.080 | 45.844 | **0.000**** | 0.510 | 0.584 | 0.449 | 0.013 | 1.014 | 0.320 | 0.023 | 6.957 | **0.011*** | 0.137 | 0.843 | 0.363 | 0.019 |
| *CV _Stride duration_ (%)* | 1.712 | 0.198 | 0.037 | 0.792 | 0.378 | 0.018 | 70.981 | **0.000**** | 0.617 | 0.026 | 0.873 | 0.001 | 0.172 | 0.680 | 0.004 | 1.135 | 0.293 | 0.025 | 0.049 | 0.826 | 0.001 |
| *CV _Stand duration_ (%)* | 0.406 | 0.527 | 0.009 | 0.262 | 0.611 | 0.006 | 57.453 | **0.000**** | 0.566 | 0.058 | 0.811 | 0.001 | 0.640 | 0.428 | 0.014 | 4.236 | **0.046*** | 0.088 | 0.049 | 0.826 | 0.001 |
| *CV _Swing duration_ (%)* | 1.420 | 0.240 | 0.031 | 0.013 | 0.910 | 0.000 | 61.838 | **0.000**** | 0.584 | 0.165 | 0.686 | 0.004 | 0.094 | 0.761 | 0.002 | 3.041 | **0.088^†^** | 0.065 | 0.143 | 0.707 | 0.003 |
| *CV _Stand phase_ (%)* | 2.916 | **0.095^†^** | 0.062 | 0.132 | 0.718 | 0.003 | 61.296 | **0.000**** | 0.582 | 0.338 | 0.564 | 0.008 | 0.038 | 0.846 | 0.001 | 3.145 | **0.083^†^** | 0.067 | 0.010 | 0.921 | 0.000 |
| *CV _Swing phase_ (%)* | 3.927 | **0.054^†^** | 0.082 | 0.289 | 0.594 | 0.007 | 61.243 | **0.000**** | 0.582 | 1.674 | 0.203 | 0.037 | 0.004 | 0.950 | 0.000 | 3.634 | **0.063^†^** | 0.076 | 0.015 | 0.903 | 0.000 |
| *CV _Cadence_ (%)* | 0.287 | 0.595 | 0.006 | 0.009 | 0.927 | 0.000 | 52.711 | **0.000**** | 0.545 | 0.809 | 0.373 | 0.018 | 1.777 | 0.189 | 0.039 | 2.448 | 0.125 | 0.053 | 0.373 | 0.545 | 0.008 |
|  |  |  |  |  |  |  |  |  |  |  |  |  |  |  |  |  |  |  |  |  |  |
| Cognitive Performance |  |  |  |  |  |  |  |  |  |  |  |  |  |  |  |  |  |  |  |  |  |
|  |  |  |  |  |  |  |  |  |  |  |  |  |  |  |  |  |  |  |  |  |  |
| *Single-Task* |  |  |  |  |  |  |  |  |  |  |  |  |  |  |  |  |  |  |  |  |  |
| *WLG* | 2.189 | 0.146 | 0.047 | 1.737 | 0.194 | 0.038 | 4.787 | **0.034*** | 0.098 | 4.389 | **0.042*** | 0.091 | 0.014 | 0.906 | 0.000 | 7.205 | **0.010**** | 0.141 | 0.234 | 0.631 | 0.005 |
| *Arithmetic task* | 0.425 | 0.518 | 0.010 | 0.208 | 0.651 | 0.005 | 4.015 | **0.051^†^** | 0.084 | 1.664 | 0.204 | 0.036 | 0.325 | 0.571 | 0.007 | 0.000 | 0.998 | 0.000 | 0.346 | 0.559 | 0.008 |
| *Stroop-Task* | 0.207 | 0.651 | 0.005 | 1.345 | 0.252 | 0.030 | 1.399 | 0.243 | 0.031 | 0.001 | 0.978 | 0.000 | 0.000 | 0.994 | 0.000 | 1.051 | 0.311 | 0.023 | 0.052 | 0.820 | 0.001 |
|  |  |  |  |  |  |  |  |  |  |  |  |  |  |  |  |  |  |  |  |  |  |
| *Dual-Task* |  |  |  |  |  |  |  |  |  |  |  |  |  |  |  |  |  |  |  |  |  |
| *WLG* | 0.304 | 0.584 | 0.007 | 0.050 | 0.825 | 0.001 | 9.046 | **0.004**** | 0.171 | 0.470 | 0.496 | 0.011 | 0.430 | 0.515 | 0.010 | 0.479 | 0.492 | 0.011 | 2.122 | 0.152 | 0.046 |
| *Arithmetic task* | 0.308 | 0.581 | 0.007 | 1.527 | 0.223 | 0.034 | 3.211 | **0.080^†^** | 0.068 | 1.547 | 0.220 | 0.034 | 0.083 | 0.775 | 0.002 | 0.025 | 0.876 | 0.001 | 0.387 | 0.537 | 0.009 |
| *Stroop-Task* | 0.449 | 0.506 | 0.010 | 0.689 | 0.411 | 0.015 | 3.085 | **0.086^†^** | 0.066 | 0.277 | 0.601 | 0.006 | 1.337 | 0.254 | 0.029 | 0.084 | 0.773 | 0.002 | 2.596 | 0.114 | 0.056 |
|  |  |  |  |  |  |  |  |  |  |  |  |  |  |  |  |  |  |  |  |  |  |
| Visual analog scale |  |  |  |  |  |  |  |  |  |  |  |  |  |  |  |  |  |  |  |  |  |
|  |  |  |  |  |  |  |  |  |  |  |  |  |  |  |  |  |  |  |  |  |  |
| *Stress* |  |  |  |  |  |  |  |  |  |  |  |  |  |  |  |  |  |  |  |  |  |
| *WLG* | 0.166 | 0.686 | 0.004 | 1.081 | 0.304 | 0.024 | 0.891 | 0.350 | 0.020 | 0.483 | 0.491 | 0.011 | 0.296 | 0.589 | 0.007 | 0.897 | 0.349 | 0.020 | 3.029 | **0.089^†^** | 0.064 |
| *Arithmetic task* | 0.014 | 0.905 | 0.000 | 0.514 | 0.477 | 0.012 | 0.722 | 0.400 | 0.016 | 0.266 | 0.609 | 0.006 | 1.268 | 0.266 | 0.028 | 0.241 | 0.626 | 0.005 | 1.880 | 0.177 | 0.041 |
| *Stroop-Task* | 1.587 | 0.214 | 0.035 | 0.239 | 0.628 | 0.005 | 2.104 | 0.154 | 0.046 | 2.806 | 0.101 | 0.060 | 6.995 | **0.011*** | 0.137 | 0.419 | 0.521 | 0.009 | 0.008 | 0.931 | 0.000 |
|  |  |  |  |  |  |  |  |  |  |  |  |  |  |  |  |  |  |  |  |  |  |
| *Prioritization/ Attention*  *(motor task – cognitive task)* |  |  |  |  |  |  |  |  |  |  |  |  |  |  |  |  |  |  |  |  |  |
| *WLG* | 0.006 | 0.938 | 0.000 | 2.678 | 0.109 | 0.057 | 0.134 | 0.716 | 0.003 | 0.290 | 0.593 | 0.007 | 3.049 | **0.088^†^** | 0.065 | 0.378 | 0.542 | 0.009 | 0.150 | 0.701 | 0.003 |
| *Arithmetic task* | 0.021 | 0.885 | 0.000 | 0.357 | 0.553 | 0.008 | 0.007 | 0.933 | 0.000 | 0.170 | 0.682 | 0.004 | 0.228 | 0.636 | 0.005 | 0.324 | 0.572 | 0.007 | 1.481 | 0.230 | 0.033 |
| *Stroop-Task* | 0.017 | 0.898 | 0.000 | 0.014 | 0.908 | 0.000 | 0.000 | 0.996 | 0.000 | 0.066 | 0.799 | 0.001 | 1.815 | 0.185 | 0.040 | 0.244 | 0.641 | 0.006 | 0.103 | 0.750 | 0.002 |
|  |  |  |  |  |  |  |  |  |  |  |  |  |  |  |  |  |  |  |  |  |  |
| Intervention Task |  |  |  |  |  |  |  |  |  |  |  |  |  |  |  |  |  |  |  |  |  |
|  |  |  |  |  |  |  |  |  |  |  |  |  |  |  |  |  |  |  |  |  |  |
| *Stroop-Task* |  |  |  |  |  |  |  |  |  |  |  |  |  |  |  |  |  |  |  |  |  |
| *Number of tasks* | - | *-* | - | 2.066 | **0.073^†^** | 0.064 | 33.385 | **0.000**** | 0.527 | - | *-* | - | - | *-* | - | 2.102 | **0.068^†^** | 0.065 | - | *-* | - |
| *Reaction time* | - | *-* | - | 3.467 | **0.005**** | 0.104 | 7.827 | **0.009**** | 0.207 | - | *-* | - | - | *-* | - | 1.111 | 0.357 | 0.036 | - | *-* | - |
| *Accuracy* | - | *-* | - | 1.049 | 0.391 | 0.034 | 0.845 | 0.365 | 0.027 | - | *-* | - | - | *-* | - | 0.414 | 0.839 | 0.014 | - | *-* | - |
|  |  |  |  |  |  |  |  |  |  |  |  |  |  |  |  |  |  |  |  |  |  |
| Trial Making Test (TMT) |  |  |  |  |  |  |  |  |  |  |  |  |  |  |  |  |  |  |  |  |  |
|  |  |  |  |  |  |  |  |  |  |  |  |  |  |  |  |  |  |  |  |  |  |
| *TMT-A* | 2.757 | 0.104 | 0.060 | 0.013 | 0.911 | 0.000 | 56.322 | **0.000**** | 0.567 | 0.118 | 0.733 | 0.003 | 0.317 | 0.576 | 0.007 | 0.339 | 0.564 | 0.008 | 2.021 | 0.162 | 0.045 |
| *TMT-B* | 1.506 | 0.226 | 0.034 | 2.359 | 0.132 | 0.052 | 42.087 | **0.000**** | 0.495 | 0.067 | 0.797 | 0.002 | 0.498 | 0.484 | 0.011 | 6.450 | **0.015*** | 0.130 | 0.037 | 0.848 | 0.001 |
| *TMT (B-A)* | 0.297 | 0.588 | 0.007 | 1.912 | 0.174 | 0.043 | 21.605 | **0.000**** | 0.334 | 0.027 | 0.871 | 0.001 | 1.563 | 0.218 | 0.035 | 3.662 | **0.062^†^** | 0.078 | 0.317 | 0.576 | 0.007 |
|  |  |  |  |  |  |  |  |  |  |  |  |  |  |  |  |  |  |  |  |  |  |

Tab.S11. Spatio-temporal gait parameters of the young and old participants during single- and dual-task walking - *manuscript-specific gait parameters*.

| **Parameter** | **Condition** |  | **YOUNG** | | | | | | | | |
| --- | --- | --- | --- | --- | --- | --- | --- | --- | --- | --- | --- |
|  |  |  |  | | | | | | | | |
|  |  |  | **Pre** | | | |  | **Post** | | | |
|  |  |  |  | | | |  |  | | | |
|  |  |  | **ST** | **WLG** | **AT** | **STR** |  | **ST** | **WLG** | **AT** | **STR** |
|  |  |  |  |  |  |  |  |  |  |  |  |
| Step width (m) | **Stroop** |  | 0.108 ± 0.029 | 0.123 ± 0.027 | 0.121 ± 0.034 | 0.115 ± 0.035 |  | 0.102 ± 0.033 | 0.113 ± 0.033 | 0.113 ± 0.034 | 0.108 ± 0.035 |
|  | **Reading** |  | 0.107 ± 0.029 | 0.120 ± 0.032 | 0.121 ± 0.030 | 0.116 ± 0.031 |  | 0.103 ± 0.031 | 0.113 ± 0.029 | 0.112 ± 0.030 | 0.107 ± 0.031 |
|  |  |  |  |  |  |  |  |  |  |  |  |
| Step length (m) | **Stroop** |  | 0.663 ± 0.065 | 0.662 ± 0.065 | 0.662 ± 0.065 | 0.659 ± 0.064 |  | 0.664 ± 0.064 | 0.665 ± 0.063 | 0.664 ± 0.062 | 0.665 ± 0.063 |
|  | **Reading** |  | 0.660 ± 0.064 | 0.662 ± 0.066 | 0.665 ± 0.065 | 0.661 ± 0.065 |  | 0.665 ± 0.067 | 0.666 ± 0.065 | 0.667 ± 0.064 | 0.664 ± 0.065 |
|  |  |  |  |  |  |  |  |  |  |  |  |
| Stride length (m) | **Stroop** |  | 1.411 ± 0.139 | 1.406 ± 0.138 | 1.406 ± 0.138 | 1.397 ± 0.136 |  | 1.408 ± 0.137 | 1.409 ± 0.134 | 1.409 ± 0.134 | 1.406 ± 0.134 |
|  | **Reading** |  | 1.402 ± 0.135 | 1.404 ± 0.141 | 1.411 ± 0.137 | 1.402 ± 0.137 |  | 1.411 ± 0.139 | 1.413 ± 0.138 | 1.416 ± 0.136 | 1.407 ± 0.137 |
|  |  |  |  |  |  |  |  |  |  |  |  |
|  |  |  | ***OLD*** | | | | | | | | |
|  |  |  |  |  |  |  |  |  |  |  |  |
| Step width (m) | **Stroop** |  | 0.122 ± 0.042 | 0.143 ± 0.047 | 0.141 ± 0.046 | 0.138 ± 0.043 |  | 0.113 ± 0.038 | 0.134 ± 0.047 | 0.131 ± 0.044 | 0.127 ± 0.042 |
|  | **Reading** |  | 0.121 ± 0.044 | 0.142 ± 0.050 | 0.141 ± 0.047 | 0.139 ± 0.047 |  | 0.118 ± 0.042 | 0.136 ± 0.047 | 0.133 ± 0.045 | 0.129 ± 0.045 |
|  |  |  |  |  |  |  |  |  |  |  |  |
| Step length (m) | **Stroop** |  | 0.516 ± 0.060 | 0.520 ± 0.063 | 0.516 ± 0.066 | 0.507 ± 0.064 |  | 0.521 ± 0.060 | 0.523 ± 0.065 | 0.520 ± 0.065 | 0.512 ± 0.063 |
|  | **Reading** |  | 0.508 ± 0.062 | 0.513 ± 0.070 | 0.508 ± 0.077 | 0.499 ± 0.073 |  | 0.522 ± 0.066 | 0.524 ± 0.067 | 0.518 ± 0.067 | 0.512 ± 0.065 |
|  |  |  |  |  |  |  |  |  |  |  |  |
| Stride length (m) | **Stroop** |  | 1.077 ± 0.127 | 1.086 ± 0.136 | 1.077 ± 0.139 | 1.058 ± 0.135 |  | 1.087 ± 0.130 | 1.095 ± 0.140 | 1.086 ± 0.136 | 1.067 ± 0.135 |
|  | **Reading** |  | 1.070 ± 0.135 | 1.076 ± 0.147 | 1.067 ± 0.154 | 1.046 ± 0.149 |  | 1.092 ± 0.137 | 1.095 ± 0.140 | 1.083 ± 0.140 | 1.068 ± 0.137 |
|  |  |  |  |  |  |  |  |  |  |  |  |

Data are presented as mean ± standard deviation. ST, Single-Task; WLG, Word list generation task; AT, Arithmetic task; STR, Stroop-task.

Tab.S12. Tab.S12: Coefficient of variation (CV) for the spatio-temporal gait parameters of the young and old participants during single- and dual-task walking - *manuscript-specific gait parameters*.

| **Parameter** | **Condition** |  | **YOUNG** | | | | | | | | |
| --- | --- | --- | --- | --- | --- | --- | --- | --- | --- | --- | --- |
|  |  |  |  | | | | | | | | |
|  |  |  | **Pre** | | | |  | **Post** | | | |
|  |  |  |  | | | |  |  | | | |
|  |  |  | **ST** | **WLG** | **AT** | **STR** |  | **ST** | **WLG** | **AT** | **STR** |
|  |  |  |  |  |  |  |  |  |  |  |  |
| CV _Step width_ (%) | **Stroop** |  | 17.94 ± 7.06 | 17.98 ± 7.28 | 18.03 ± 8.95 | 17.88 ± 7.80 |  | 20.87 ± 9.68 | 20.80 ± 10.54 | 19.88 ± 10.41 | 20.19 ± 8.40 |
|  | **Reading** |  | 17.72 ± 8.32 | 19.94 ± 10.36 | 17.26 ± 6.79 | 17.10 ± 7.52 |  | 20.29 ± 10.68 | 20.67 ± 9.63 | 19.10 ± 9.68 | 19.43 ± 8.23 |
|  |  |  |  |  |  |  |  |  |  |  |  |
| CV _Step length_ (%) | **Stroop** |  | 1.70 ± 0.40 | 1.90 ± 0.56 | 1.87 ± 0.51 | 1.72 ± 0.47 |  | 1.62 ± 0.39 | 1.67 ± 0.38 | 1.63 ± 0.37 | 1.60 ± 0.40 |
|  | **Reading** |  | 1.68 ± 0.38 | 1.89 ± 0.51 | 1.74 ± 0.46 | 1.66 ± 0.46 |  | 1.59 ± 0.32 | 1.73 ± 0.35 | 1.61 ± 0.38 | 1.60 ± 0.42 |
|  |  |  |  |  |  |  |  |  |  |  |  |
| CV _Stride length_ (%) | **Stroop** |  | 1.34 ± 0.29 | 1.54 ± 0.45 | 1.53 ± 0.44 | 1.31 ± 0.35 |  | 1.33 ± 0.32 | 1.37 ± 0.31 | 1.27 ± 0.25 | 1.20 ± 0.27 |
|  | **Reading** |  | 1.32 ± 0.25 | 1.57 ± 0.42 | 1.38 ± 0.34 | 1.24 ± 0.33 |  | 1.32 ± 0.28 | 1.38 ± 0.25 | 1.31 ± 0.34 | 1.24 ± 0.32 |
|  |  |  |  |  |  |  |  |  |  |  |  |

|  |  |  | **OLD** | | | | | | | | |
| --- | --- | --- | --- | --- | --- | --- | --- | --- | --- | --- | --- |
|  |  |  |  |  |  |  |  |  |  |  |  |
| CV _Step width_ (%) | **Stroop** |  | 18.81 ± 12.97 | 18.10 ± 10.83 | 16.94 ± 12.48 | 17.43 ± 12.49 |  | 20.93 ± 15.22 | 20.57 ± 17.83 | 19.33 ± 15.86 | 20.33 ± 16.97 |
|  | **Reading** |  | 21.44 ± 15.92 | 19.46 ± 17.87 | 18.18 ± 14.87 | 19.45 ± 16.18 |  | 27.90 ± 47.99 | 21.31 ± 18.13 | 18.30 ± 14.98 | 21.65 ± 23.60 |
|  |  |  |  |  |  |  |  |  |  |  |  |
| CV _Step length_ (%) | **Stroop** |  | 3.58 ± 0.95 | 4.19 ± 1.48 | 4.37 ± 1.53 | 4.16 ± 1.42 |  | 3.67 ± 1.13 | 4.01 ± 1.57 | 3.96 ± 1.16 | 3.68 ± 1.14 |
|  | **Reading** |  | 3.80 ± 1.23 | 4.50 ± 1.53 | 4.44 ± 1.63 | 4.61 ± 2.02 |  | 3.43 ± 1.04 | 4.57 ± 2.67 | 3.94 ± 1.31 | 4.05 ± 1.42 |
|  |  |  |  |  |  |  |  |  |  |  |  |
| CV _Stride length_ (%) | **Stroop** |  | 2.63 ± 0.62 | 3.13 ± 1.05 | 3.31 ± 1.36 | 2.95 ± 1.01 |  | 2.76 ± 0.76 | 2.74 ± 0.86 | 2.89 ± 0.86 | 2.67 ± 0.79 |
|  | **Reading** |  | 2.75 ± 0.78 | 3.27 ± 1.01 | 3.26 ± 1.18 | 3.24 ± 1.43 |  | 2.55 ± 0.75 | 2.86 ± 0.89 | 2.88 ± 0.85 | 2.83 ± 1.04 |
|  |  |  |  |  |  |  |  |  |  |  |  |

|  |  |  |  |  |  |  |  |  |  |  |  |
| --- | --- | --- | --- | --- | --- | --- | --- | --- | --- | --- | --- |

CV, coefficient of variation. ST, Single-Task; WLG, Word list generation task; AT, Arithmetic task; STR, Stroop-task. Data are presented as mean ± standard deviation.

Tab.S13. Statistical outcomes (ANOVA) - *manuscript-specific gait parameters.*

|  |  | | |  | | |  | | |  | | |  | | |  | | |  | | |
| --- | --- | --- | --- | --- | --- | --- | --- | --- | --- | --- | --- | --- | --- | --- | --- | --- | --- | --- | --- | --- | --- |
|  | **Condition** | | | **Time** | | | **Group** | | | **Condition x Time** | | | **Condition x Group** | | | **Time x Group** | | | **Condition x Time x Group** | | |
|  | **F** | ***P*** | **Ƞ_p_^2^** | **F** | ***P*** | **Ƞ_p_^2^** | **F** | ***P*** | **Ƞ_p_^2^** | **F** | ***P*** | **Ƞ_p_^2^** | **F** | ***P*** | **Ƞ_p_^2^** | **F** | ***P*** | **Ƞ_p_^2^** | **F** | ***P*** | **Ƞ_p_^2^** |
| Gait Parameter |  |  |  |  |  |  |  |  |  |  |  |  |  |  |  |  |  |  |  |  |  |
|  |  |  |  |  |  |  |  |  |  |  |  |  |  |  |  |  |  |  |  |  |  |
| *Single-Task* |  |  |  |  |  |  |  |  |  |  |  |  |  |  |  |  |  |  |  |  |  |
|  |  |  |  |  |  |  |  |  |  |  |  |  |  |  |  |  |  |  |  |  |  |
| *Step width (m)* | 3.863 | **0.056^†^** | 0.081 | 6.417 | **0.015*** | 0.127 | 0.753 | 0.390 | 0.017 | 2.581 | 0.115 | 0.055 | 0.008 | 0.931 | 0.000 | 0.866 | 0.357 | 0.019 | 1.857 | 0.180 | 0.040 |
| *Step length (m)* | 0.411 | 0.525 | 0.009 | 6.932 | **0.012*** | 0.136 | 58.219 | **0.000**** | 0.570 | 3.494 | **0.068^†^** | 0.074 | 0.137 | 0.713 | 0.003 | 6.033 | **0.018*** | 0.121 | 1.312 | 0.258 | 0.029 |
| *Stride length (m)* | 1.512 | 0.225 | 0.033 | 1.902 | 0.175 | 0.041 | 64.293 | **0.000**** | 0.594 | 1.871 | 0.178 | 0.041 | 0.240 | 0.627 | 0.005 | 5.452 | **0.024*** | 0.110 | 0.059 | 0.810 | 0.001 |
|  |  |  |  |  |  |  |  |  |  |  |  |  |  |  |  |  |  |  |  |  |  |
| *CV _Step width_ (%)* | 2.209 | 0.144 | 0.048 | 2.393 | 0.129 | 0.052 | 1.037 | 0.314 | 0.023 | 1.317 | 0.257 | 0.029 | 2.435 | 0.126 | 0.052 | 0.38 | 0.541 | 0.009 | 0.748 | 0.392 | 0.017 |
| *CV _Step length_ (%)* | 0.483 | 0.491 | 0.011 | 0.001 | 0.980 | 0.000 | 87.904 | **0.000**** | 0.666 | 0.024 | 0.876 | 0.001 | 0.011 | 0.915 | 0.000 | 0.111 | 0.741 | 0.003 | 2.995 | **0.091^†^** | 0.064 |
| *CV _Stride length_ (%)* | 1.847 | 0.181 | 0.040 | 0.330 | 0.569 | 0.007 | 103.175 | **0.000**** | 0.701 | 0.143 | 0.707 | 0.003 | 0.240 | 0.627 | 0.005 | 0.042 | 0.839 | 0.001 | 2.023 | 0.162 | 0.044 |
|  |  |  |  |  |  |  |  |  |  |  |  |  |  |  |  |  |  |  |  |  |  |
| Dual-Task |  |  |  |  |  |  |  |  |  |  |  |  |  |  |  |  |  |  |  |  |  |
| *WLG* |  |  |  |  |  |  |  |  |  |  |  |  |  |  |  |  |  |  |  |  |  |
|  |  |  |  |  |  |  |  |  |  |  |  |  |  |  |  |  |  |  |  |  |  |
| *Step width (m)* | 0.530 | 0.471 | 0.012 | 3.130 | **0.084^†^** | 0.066 | 2.063 | 0.158 | 0.045 | 1.944 | 0.170 | 0.042 | 0.060 | 0.807 | 0.001 | 0.082 | 0.776 | 0.002 | 0.043 | 0.837 | 0.001 |
| *Step length (m)* | 0.007 | 0.933 | 0.000 | 1.190 | 0.281 | 0.026 | 54.325 | **0.000**** | 0.553 | 0.303 | 0.585 | 0.007 | 0.635 | 0.430 | 0.014 | 0.569 | 0.455 | 0.013 | 1.506 | 0.226 | 0.033 |
| *Stride length (m)* | 0.008 | 0.928 | 0.000 | 0.198 | 0.659 | 0.004 | 58.631 | **0.000**** | 0.571 | 0.021 | 0.886 | 0.000 | 0.528 | 0.471 | 0.012 | 1.208 | 0.278 | 0.027 | 0.184 | 0.670 | 0.004 |
|  |  |  |  |  |  |  |  |  |  |  |  |  |  |  |  |  |  |  |  |  |  |
| *CV _Step width_ (%)* | 0.262 | 0.611 | 0.006 | 4.490 | **0.040*** | 0.093 | 0.126 | 0.724 | 0.003 | 3.311 | **0.076^†^** | 0.070 | 0.013 | 0.910 | 0.000 | 0.328 | 0.570 | 0.007 | 0.023 | 0.880 | 0.001 |
| *CV _Step length_ (%)* | 0.591 | 0.446 | 0.013 | 0.409 | 0.526 | 0.009 | 0.051 | 0.823 | 0.001 | 0.006 | 0.937 | 0.000 | 0.409 | 0.526 | 0.009 | 0.104 | 0.749 | 0.002 | 0.051 | 0.823 | 0.001 |
| *CV _Stride length_ (%)* | 0.569 | 0.455 | 0.013 | 1.176 | 0.284 | 0.026 | 59.737 | **0.000**** | 0.576 | 0.071 | 0.791 | 0.002 | 0.317 | 0.576 | 0.007 | 1.670 | 0.203 | 0.037 | 0.002 | 0.968 | 0.000 |
|  |  |  |  |  |  |  |  |  |  |  |  |  |  |  |  |  |  |  |  |  |  |
| Dual-Task |  |  |  |  |  |  |  |  |  |  |  |  |  |  |  |  |  |  |  |  |  |
| *Arithmetic task* |  |  |  |  |  |  |  |  |  |  |  |  |  |  |  |  |  |  |  |  |  |
|  |  |  |  |  |  |  |  |  |  |  |  |  |  |  |  |  |  |  |  |  |  |
| *Step width (m)* | 1.062 | 0.308 | 0.024 | 13.841 | **0.001**** | 0.239 | 1.563 | 0.218 | 0.034 | 0.165 | 0.687 | 0.004 | 0.001 | 0.980 | 0.000 | 0.691 | 0.410 | 0.015 | 0.811 | 0.373 | 0.018 |
| *Step length (m)* | 0.592 | 0.446 | 0.013 | 0.335 | 0.566 | 0.008 | 58.215 | **0.000**** | 0.570 | 0.515 | 0.477 | 0.012 | 3.707 | **0.061^†^** | 0.078 | 2.339 | 0.133 | 0.050 | 0.626 | 0.433 | 0.014 |
| *Stride length (m)* | 1.241 | 0.271 | 0.027 | 0.324 | 0.572 | 0.007 | 65.135 | **0.000**** | 0.597 | 0.181 | 0.672 | 0.004 | 2.641 | 0.111 | 0.057 | 1.699 | 0.199 | 0.037 | 0.066 | 0.799 | 0.001 |
|  |  |  |  |  |  |  |  |  |  |  |  |  |  |  |  |  |  |  |  |  |  |
| *CV _Step width_ (%)* | 0.579 | 0.451 | 0.013 | 12.031 | **0.001**** | 0.215 | 0.064 | 0.801 | 0.001 | 2.271 | 0.139 | 0.049 | 0.287 | 0.595 | 0.006 | 0.001 | 0.971 | 0.000 | 2.998 | **0.090^†^** | 0.064 |
| *CV _Step length_ (%)* | 0.970 | 0.330 | 0.022 | 0.499 | 0.484 | 0.011 | 75.346 | **0.000**** | 0.631 | 0.758 | 0.389 | 0.017 | 0.036 | 0.851 | 0.001 | 3.144 | **0.083^†^** | 0.067 | 0.241 | 0.626 | 0.005 |
| *CV _Stride length_ (%)* | 0.578 | 0.451 | 0.013 | 0.263 | 0.610 | 0.006 | 70.688 | **0.000**** | 0.616 | 0.340 | 0.563 | 0.008 | 0.006 | 0.939 | 0.000 | 2.003 | 0.164 | 0.044 | 0.310 | 0.580 | 0.007 |
|  |  |  |  |  |  |  |  |  |  |  |  |  |  |  |  |  |  |  |  |  |  |
| Dual-Task |  |  |  |  |  |  |  |  |  |  |  |  |  |  |  |  |  |  |  |  |  |
| *Stroop-Task* |  |  |  |  |  |  |  |  |  |  |  |  |  |  |  |  |  |  |  |  |  |
|  |  |  |  |  |  |  |  |  |  |  |  |  |  |  |  |  |  |  |  |  |  |
| *Step width (m)* | 3.722 | **0.060^†^** | 0.078 | 9.295 | **0.004**** | 0.174 | 2.284 | 0.138 | 0.049 | 0.021 | 0.885 | 0.000 | 0.037 | 0.847 | 0.001 | 1.617 | 0.210 | 0.035 | 0.785 | 0.380 | 0.018 |
| *Step length (m)* | 0.307 | 0.582 | 0.007 | 0.121 | 0.730 | 0.003 | 63.377 | **0.000**** | 0.590 | 0.000 | 0.988 | 0.000 | 1.282 | 0.264 | 0.028 | 1.224 | 0.275 | 0.027 | 4.021 | **0.051^†^** | 0.084 |
| *Stride length (m)* | 0.591 | 0.446 | 0.013 | 0.054 | 0.818 | 0.001 | 70.007 | **0.000**** | 0.614 | 0.110 | 0.742 | 0.002 | 0.774 | 0.384 | 0.017 | 1.019 | 0.318 | 0.023 | 3.098 | **0.085^†^** | 0.066 |
|  |  |  |  |  |  |  |  |  |  |  |  |  |  |  |  |  |  |  |  |  |  |
| *CV _Step width_ (%)* | 1.966 | 0.168 | 0.043 | 5.590 | **0.023*** | 0.113 | 0.431 | 0.515 | 0.010 | 0.019 | 0.890 | 0.000 | 3.810 | **0.057^†^** | 0.080 | 0.232 | 0.632 | 0.005 | 0.159 | 0.692 | 0.004 |
| *CV _Step length_ (%)* | 1.996 | 0.165 | 0.043 | 0.015 | 0.902 | 0.000 | 59.157 | **0.000**** | 0.573 | 0.009 | 0.925 | 0.000 | 3.184 | **0.081^†^** | 0.067 | 5.839 | **0.020*** | 0.117 | 0.407 | 0.527 | 0.009 |
| *CV _Stride length_ (%)* | 0.988 | 0.326 | 0.022 | 0.317 | 0.576 | 0.007 | 56.103 | **0.000**** | 0.560 | 0.043 | 0.836 | 0.001 | 1.303 | 0.260 | 0.029 | 6.379 | **0.015*** | 0.127 | 0.994 | 0.324 | 0.022 |
|  |  |  |  |  |  |  |  |  |  |  |  |  |  |  |  |  |  |  |  |  |  |


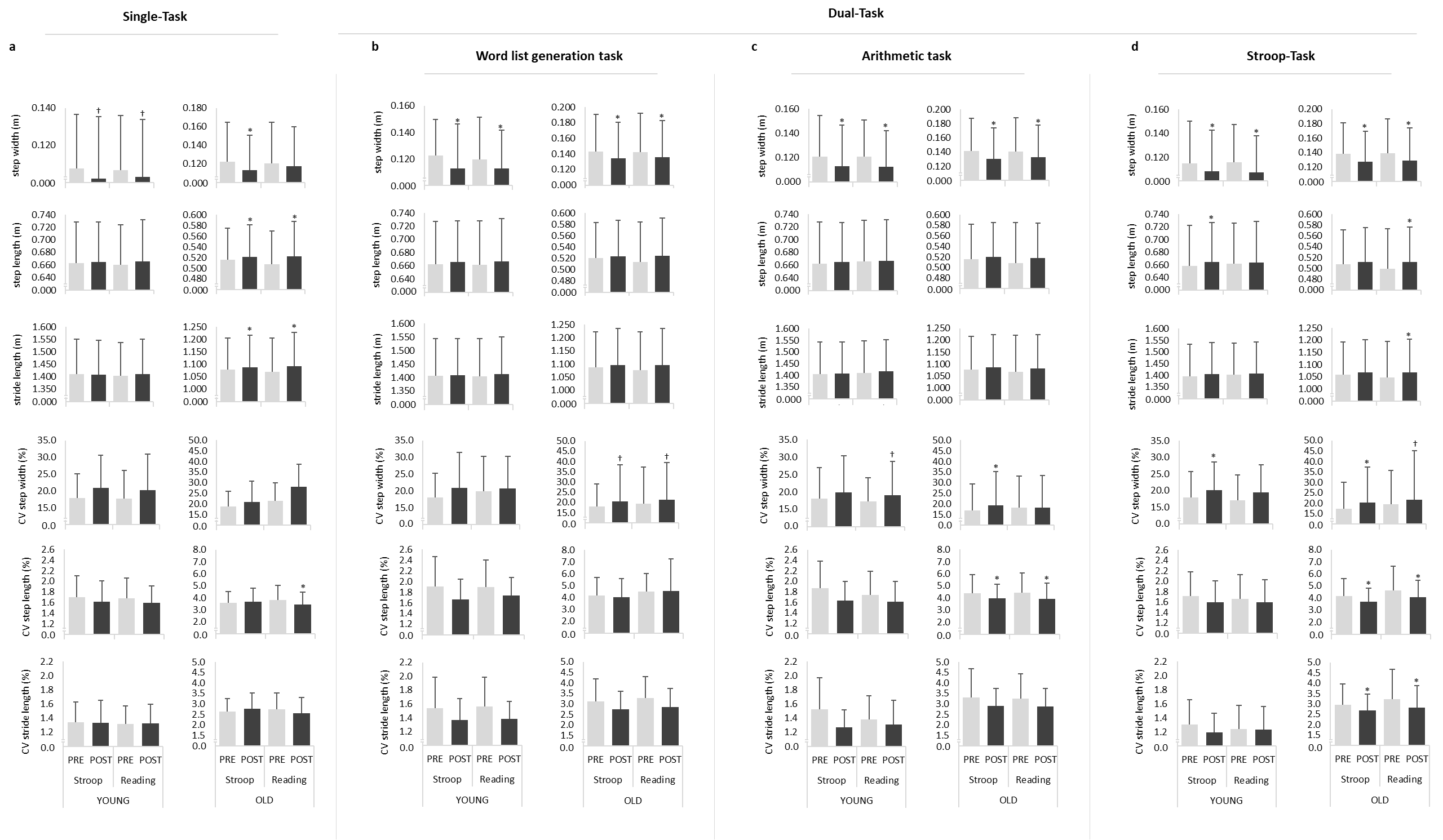


Fig.S1. Spatio-temporal gait parameters (step width, step length and stride length) and their respective coefficient of variation (CV) during single and dual-task walking. (**a**) and Dual-Task: Word list generation task (**b**), Arithmetic task (**c**) and Stroop-Task (**d**) for the young and old participants recorded before (PRE) and after (POST) the intervention (Stroop intervention task (Stroop), reading control task (Reading)). * *P* ≤ 0.050, † *P* ≤ 0.100.
